# Promiscuous activity of C-acyltransferase from *Pseudomonas protegens*: synthesis of acetanilides in aqueous buffer†
†Electronic supplementary information (ESI) available. See DOI: 10.1039/c8cc00290h


**DOI:** 10.1039/c8cc00290h

**Published:** 2018-03-19

**Authors:** Anna Żądło-Dobrowolska, Nina G. Schmidt, Wolfgang Kroutil

**Affiliations:** a Institute of Chemistry , University of Graz , NAWI Graz , BioTechMed Graz , Harrachgasse 21/3 , Graz , Austria . Email: wolfgang.kroutil@uni-graz.at; b Austrian Centre of Industrial Biotechnology , acib GmbH , Graz , Austria

## Abstract

Amide bond formation has considerable significance in synthetic chemistry. Although the C-acyltransferase from *Pseudomonas protegens* has been found to catalyze C–C bond formation in nature as well as in *in vitro* experiments with non-natural substrates, it is now shown that the enzyme is also able to catalyze amide formation using aniline derivatives as substrates with promiscuous activity. Importantly, the amide formation was enabled in aqueous buffer. Identifying phenyl acetate as the most suitable acetyl donor, the products were obtained with up to >99% conversion and up to 99% isolated yield.

## Abstract 2

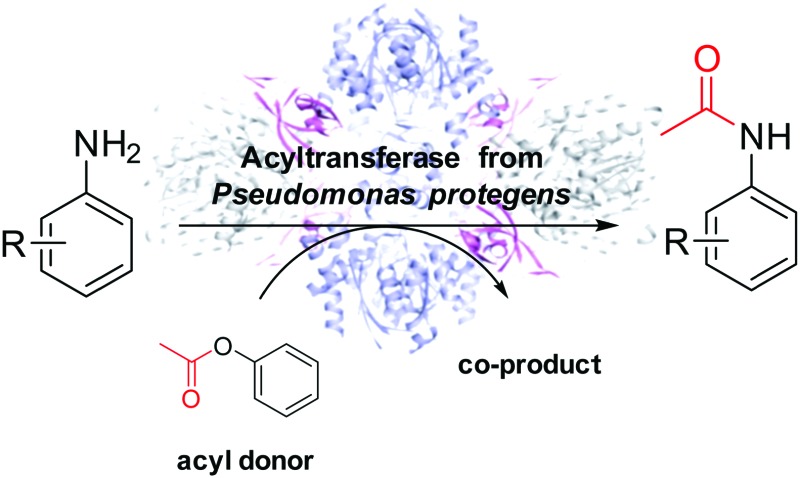
A C-acyltransferase was found to show promiscuous activity catalyzing C–N bond formation in aqueous buffer instead of C–C bond formation.

## 

The amide moiety is the key functional group in a wide variety of biological and synthetic structures, such as peptides, polymers, pharmaceuticals and pesticides.^1^ Formally, an amide bond is formed during the condensation of an amine and carboxylic acid with the release of one molecule of water. Due to the high activation barrier, synthetic amides are mainly produced by employing activated acid derivatives, such as acid chlorides and anhydrides.^2^ Although these methods tend to be highly effective, they suffer from several drawbacks, such as possible racemization, poor atom economy, the possible toxic nature of organic solvents and coupling reagents. As a consequence, amide bond formation has been identified as one of the most important synthetic processes to be improved.^1^,^3^ Furthermore, due to the increasing demands for green chemistry procedures,^4^ a direct, efficient, environmentally friendly catalytic process is of high importance.^5^

Various types of biocatalysts such as proteases transform amides and catalyse in their natural physiological role amide bond hydrolysis in peptide substrates.^6^ Due to the principle reversibility of catalysis and when the thermodynamic barrier (*e.g.* using an excess of water) can be overcome then the equilibrium can be shifted to favour synthesis over hydrolysis. Numerous proteases were reported to catalyse amide bond formation in nearly anhydrous organic solvents^7^ or water mimetics.^8^ However, limitations including the rather strict specificities and relative instability in anhydrous environments inspired the search for non-protease catalysts. Due to the similarities in the mechanism employing nucleophilic serine at the active site, lipases and esterases were also found to show promiscuous amidase activity.^9^ While many biocatalytic methods in low-water media have been developed, few cases have been reported concerning amide bond formation in aqueous media.^10^ In addition to hydrolases, a few other enzymes have been described to transfer the acyl group from acyl donors to amine acceptors under physiological conditions. For instance, penicillin acylase was applied for dipeptide synthesis^11^ as well as for enantioselective acylation of various amines.^12^ Peptiligase was reported as an efficient catalyst in peptide synthesis and cyclization.^13^ Also an acyltransferase originating from *Mycobacterium smegmatis* (*Ms*AcT) was shown to catalyse acylation of aliphatic amines in water.^14^

An acyltransferase from *Pseudomonas protegens* (*Pp*ATaseCH) was reported to perform C–C bond formation in the biosynthesis of the antibiotically active polyketide 2,4-diacetylphloroglucinol (DAPG), excreted by several plant-associated *Pseudomonas* sp. and *Pseudomonas fluorescens* ssp.^15^ Unlike most other acyltransferases,^16^ this heterotrimeric complex acts independently of cofactors, CoA or ATP. The multi-component ATase from *Pseudomonas protegens* catalyses also C–C bond formation when transferring an acetyl moiety from a non-natural donor substrate such as isopropenyl acetate to an electron-rich phenolic acceptor in a Friedel–Crafts-type acetylation reaction.^17^ Here we report that the enzyme shows chemical reaction promiscuity^18^ catalysing also amide formation.

Since in previous studies mainly resorcinol derivatives were investigated as acetyl acceptors leading to C–C bond formation,^17^ the substrate scope was extended by testing a substrate bearing one amino group instead of one of the hydroxyl groups in resorcinol, a substrate undergoing C–C bond formation. Indeed, 3-aminophenol (**1a**) was accepted as the substrate when using 2,4-diacetylphloroglucinol (DAPG, **4**) as the acetyl donor (Scheme 1). However, instead of the expected C–C bond formation the enzyme performed *N*-acetylation, thus *N*-(3-hydroxyphenyl)acetamide (**2a**) was formed as the only product instead of the expected 1-(2-amino-4-hydroxyphenyl)ethan-1-one (**3**, Scheme 1), which would have been formed *via* C–C bond formation.^17^ Thus, in the observed reaction the enzyme breaks a C–C bond in the acetyl donor and enables subsequently C–N bond formation. Furthermore, it has to be noted that acetylation was observed exclusively at the amino group while the phenolic alcohol was not acetylated. The selective acylation of amines in the presence of other functional groups such as phenolic alcohols is rather a difficult task. For instance, chemical acylation of such compounds leads to the formation of the corresponding amides and esters, when acid chlorides or anhydrides were employed; *N*-selectivity could only be achieved with thioesters and Cu-catalysis.^19^

**Scheme 1:**
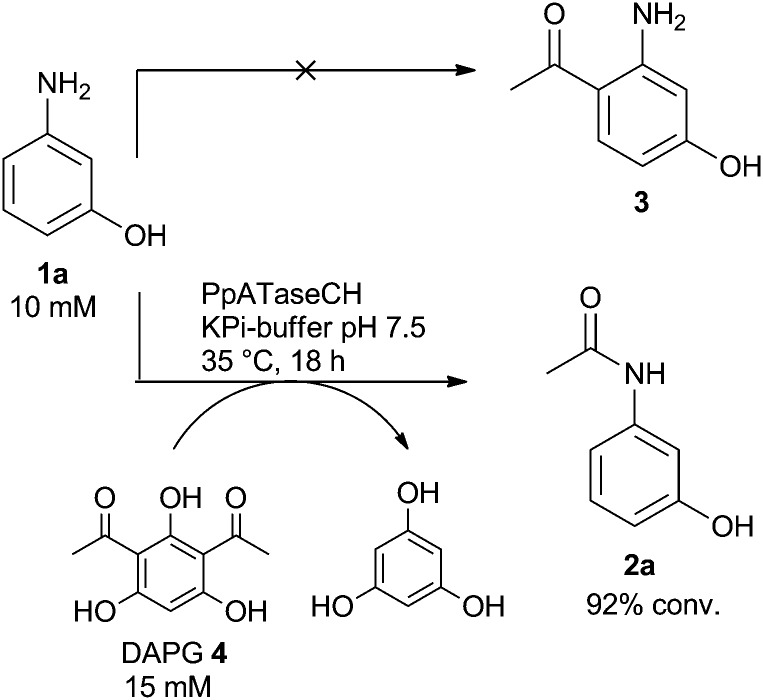
ATase catalyzes *N*-acylation of 3-aminophenol.

To gain deeper insight into the substrate spectrum, a range of aromatic amines **1b–1l** containing various functional groups was studied (Table 1). For this purpose, the substrate (10 mM) was suspended in KPi-buffer adjusted to pH 7.5, and cell-free extract containing recombinant *Pp*ATaseCH was subsequently added, followed by addition of the acetyl donor (DAPG **4**). While aniline derivatives bearing an OH group in the *para*- or *ortho*-position were not converted (substrates **1b–1d**), aniline **1e** as well as anilines with various substituents in the *para*-position (Cl, i-Pr, Et) were well accepted reaching a conversion of up to 92% within 18 hours (substrates **1f–1h**). Furthermore, anilines with an ethyl group also in the *meta*- or *ortho*-position were accepted, whereby the *para*-substituted substrate reacted the fastest followed by the *meta*-substituted ones (substrates **1h–1i**). Having a nitro-moiety in the *ortho*-position instead of the ethyl group did not lead to any detectable conversion. In addition to the mono-substituted anilines, also a di-substituted aniline (**1l**) was successfully transformed.

**Table 1:** Biocatalytic *N*-acetylation of anilines

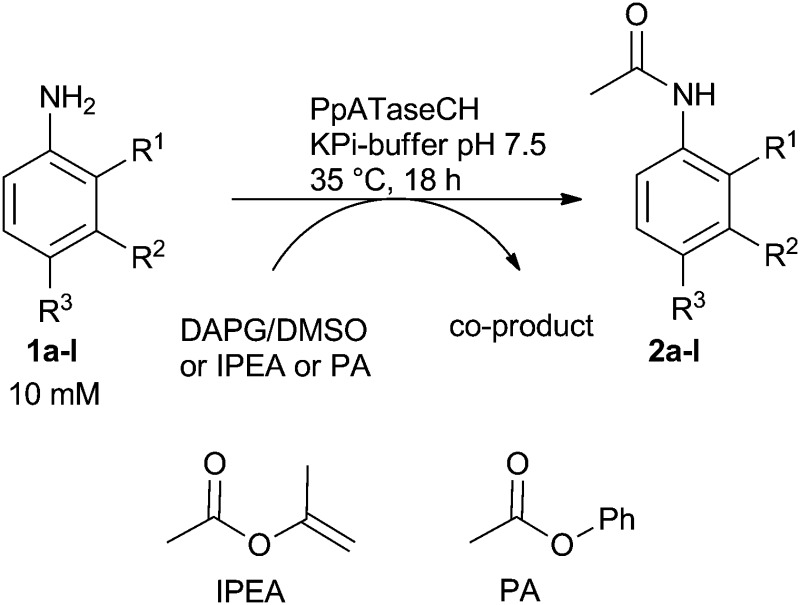							
					Acetyl donor		
					IPEA	DAPG **4**	PA
Entry	Substr.	R^1^	R^2^	R^3^	Conv. [%]	Conv. [%]	Conv. [%]
1	**1a**	H	OH	H	43	92	>99(96)
2	**1b**	H	H	OH	<1	<1	<1
3	**1c**	OH	H	H	<1	<1	<1
4	**1d**	OH	H	OH	<1	<1	<1
5	**1e**	H	H	H	63	84	>99(70)
6	**1f**	H	H	Cl	50	55	>99(91)
7	**1g**	H	H	i-Pr	66	92	>99(83)
8	**1h**	H	H	Et	47	86	>99(99)
9	**1i**	H	Et	H	33	81	>99(98)
10	**1j**	Et	H	H	12	7	18(13)
11	**1k**	NO_2_	H	H	<1	<1	<1
12	**1l**	H	CH_2_OH	Cl	12	85	98(91)

Although rather useful conversion values were achieved when using DAPG **4** as the acetyl donor, other acetyl donors were evaluated. One reason being that DAPG **4** is not commercially available and the co-product phloroglucinol generated during reaction impedes the purification. Consequently, enol esters and phenyl esters such as isopropenyl acetate (IPEA) and phenyl acetate (PA) were evaluated as donors in further experiments. For these two donors the enzyme would cleave a C–O bond.

Using IPEA as the acetyl donor with substrates **1a–1l**, 10 equivalents of IPEA were required to obtain moderate conversion, for instance, reaching 66% conversion for **1k**. In contrast, just 1.5 equivalents of PA were sufficient to observe quantitative product formation for all substrates transformed also with DAPG (Table 1, entries 1, 5–9 and 12), except for **1j** which led to lower conversion.

Thus, comparing the natural acetyl donor DAPG with the non-natural IPEA and PA revealed that PA was the most efficient acetyl source, requiring only a low excess of donor to enable the completion of the reaction for substrates **1a** and **1f–1h** under the reaction conditions tested, thus leading in all cases to higher conversion than observed with DAPG. For instance, acetylation with IPEA resulted in 50% conversion for **1f** (55% with DAPG) while >99% was achieved with PA.

Testing the transformation of aliphatic amines with the improved protocol showed that benzylamine (**1m**) or 1-octylamine was not accepted by the enzyme. This chemoselectivity may have practical application, as ATase differentiates between the aniline-NH_2_ and an aliphatic amine. To demonstrate the chemoselectivity, the acetylation of aniline derivative **1a** and benzylamine was tested in one pot simultaneously (Table 2). Using either IPEA, DAPG or PA perfect chemoselectivity toward **1a** was observed.

**Table 2:** Chemoselectivity of ATase: *N*-acetylation of aniline derivative **1a***versus* benzylamine **1m**

Entry	Donor	Conv. of **1a** [%]	Conv. of **1m** [%]	Chemoselectivity **2a** : **2m**
1	IPEA	46	n.d.	**2a** only
2	DAPG	96	n.d.	**2a** only
3	PA	100	n.d.	**2a** only

Up to now mainly acetyl and propanoyl groups have been transferred by this enzyme.17*a* The expansion of the scope of acyl donors (*e.g.* for butanoyl, benzoyl, *etc.*) by applying protein engineering is under investigation.

To test the acyltransferase *Pp*ATaseCH under the conditions previously described for the acetyltransferase from *Mycobacterium smegmatis*,^14^ an experiment was performed in CHES buffer adjusted to pH 10. However, under these conditions substrate **1a** was transformed with only 34% conversion into the corresponding acetanilide, while in phosphate buffer at pH 7.5 the transformation reached completion. This result is in accordance with our previous observations for the C–C bond forming reaction, showing that *Pp*ATaseCH displayed higher activity at pH 7.5 to 8.5, while a more alkaline pH resulted in lower enzyme activity. Since *Ms*AcT has been reported to transform only aliphatic amines, *Pp*ATaseCH complements the substrate scope by also allowing the transformation of anilines into the corresponding acetanilides.

Furthermore, lipases are also known for their amidation activity especially in organic solvents.^20^ For comparison, the *N*-acetylation of **1a** was tested in aqueous buffer using Novozym 435 as a representative lipase due to its broad substrate acceptance. However, the conversion was <1% when using either IPEA or PA after 18 h, clearly showing that Novozym 435 does not catalyze this reaction in aqueous buffer. Additionally, it was ensured by enzyme purification that no other enzyme present in *E. coli* catalyzes the observed reaction, thus the amide formation is due to the activity of *Pp*ATaseCH.

To demonstrate the applicability of the amide formation, semi-preparative transformations were performed starting with 0.25 mmol of the substrate. Anilines **1a** and **1e–j** were transformed into the corresponding acetanilides using PA as the acetyl donor within 24 h and the products were isolated with high yields (Table 1, values in brackets). The yields were in most cases comparable to the conversion obtained in analytical-scale experiments.

Finally, optimisation of the reaction system and variation of the concentration of PA for the amidation of **1a** showed the following: an excess of donor (*e.g.* 10 or 5 equivalents in 10 mM substrate) led to lower conversion than when using just 1.5 of donor PA (Table 3, entries 1–3). Actually, the equivalents of PA could be reduced even further to 1.1 equivalents (entry 4). Keeping the donor at 1.5 equivalents, the substrate concentration was increased at the given enzyme concentration up to 20 mM still leading to >99% conversion before only at 40 mM a slight drop of conv. to 95% was observed. The latter corresponds to an apparent total turnover number of TON_app_ = 5360.

**Table 3:** Intensification of the *N*-acetylation of **1a**

Entry	Substrate concentration [mM]	Donor concentration [mM]	Conv. [%]
1	10	100	80
2	10	50	86
3	10	15	>99
4	10	11	>99
5	20	30	>99
6	40	60	95

The promiscuous *N*-acetylation activity of ATase may be explained based on sequence alignment searches. The *PhlC* unit, which is responsible for the enzyme activity, can be related to the thiolase superfamily,^21^ featuring a cysteine residue at position 88 (Cys88) which aligns to the conserved active-site cysteine of thiolases. Cysteine C88 may form a thioester intermediate with the acetyl donor, which may be prone to the amine nucleophilic attack leading to the amidation product.

In summary, the acyltransferase from *Pseudomonas protegens* was found to exhibit promiscuous *N*-acetylation activity towards a broad spectrum of aniline derivatives. In contrast to previous studies in which C–C bond formation was found, the enzyme allowed the acetylation of aniline derivatives in aqueous medium. After identifying phenyl acetate as an ideal donor, excellent isolated yields of up to 99% were obtained for the *N*-acetylated anilides. The transformation was demonstrated with substrate loadings above 40 mM and a low excess of donor (1.5 eq.). The protocol involving mild reaction conditions, a simple workup procedure leading to high yields, may serve as an attractive alternative to the existing *N*-acylation methods.

We gratefully acknowledge support from the Austrian Science Fund (FWF) through a Lise Meitner Fellowship Grant M 2172-B21. NGS was financed by the Austrian FFG, BMWFJ, BMVIT, SFG, Standortagentur Tirol and ZIT through the Austrian FFG-COMET-Funding Program.

## Conflicts of interest

There are no conflicts to declare.

## Supplementary Material

Supplementary information
