# Supplementary material for: Promiscuous activity of C-acyltransferase from Pseudomonas protegens: synthesis of acetanilides in aqueous buffer
Source: Chem Commun (Camb). 2018 Mar 19;54(27):3387–90. doi: 10.1039/c8cc00290h (PMC5885802; doi:10.1039/c8cc00290h)
Supplement: Supplementary file 1 [file CC-054-C8CC00290H-s001.pdf]

Electronic Supplementary Material (ESI) for ChemComm.
This journal is © The Royal Society of Chemistry 2018

Supporting Information

Promiscuous activity of *C*-acyltransferase from *Pseudomonas protegens*: synthesis of acetanilides in buffer

Anna Żądło-Dobrowolska,[1] Nina G. Schmidt,[1,2] Wolfgang Kroutil*[1,2]

[1]Institute of Chemistry, University of Graz, NAWI Graz, BioTechMed Graz, Graz, Austria

[2]ACIB GmbH, Graz, Austria

Corresponding Authors: wolfgang.kroutil@uni-graz.at

Experimental

Materials

All starting materials were obtained from Sigma-Aldrich, Alfa Aesar or TCI-Chemicals and used as received unless stated otherwise. 1,3-Diacetyl-2,4,6-trihydroxy benzene (DAPG) was chemically synthesized as previously described.[1] Reference compounds, *N*-(3-hydroxyphenyl)acetamide, *N*-(4-ethylphenyl)acetamide, *N*-(4-isopropylphenyl)-acetamide, *N*-(4-chlorophenyl)acetamide, *N*-(2-ethylphenyl)acetamide, *N*-(3-ethylphenyl)acetamide were chemically synthesized.

Methods

TLC was carried out with pre-coated aluminum sheets (TLC Silica gel 60 F254, Merck) with detection by UV (254 nm) and/or by staining with cerium molybdate solution. GC-MS spectra were recorded with an Agilent 7890A GC-system, equipped with an Agilent 5975C mass selective detector and a HP-5 MS column (30 m × 0.25 mm × 0.25 µm). Analysis was performed according to the following parameters: injector 250 °C, constant flow 0.7 mL; temperature program: 100 °C (hold 0.5 min) 100 °C to 300 °C (10 °C min^{-1}), 300 °C (hold 2 min). Helium was used as carrier gas and EtOAc was used as solvent. ^{1}H- and ^{13}C-NMR spectra were recorded at 20 °C on a 300 MHz Bruker NMR or 500 MHz Bruker NMR. The conversions were measured at 25 °C by HPLC using a Shimadzu-Prominence liquid chromatograph, equipped with a SPD-M20A diode array detector and an achiral C18 column (Phenomenex Luna C18 (2) 100A (0.46 cm × 25 cm, 5 µm particle size). The following gradient elution with H_2O and MeCN (+TFA, 0.1 vol. %) was performed: 0-15 % MeCN (0-5 min), 15-60 % MeCN (5-22 min), 60-100 % MeCN (22-25 min), 100-0 % MeCN (25-30 min), flow rate = 1 mL min^{-1}, λ = 254 nm, injection vol. = 2 µL. Reaction products were quantified at 254 nm from the peak areas on the basis of standard curves with reference compounds. The ATase from *Pseudomonas protegens* (*Pp*ATaseCH) was overexpressed in *E. coli* BL21 (DE3) as described previously and used as cell-free extract preparations.[1]

Screening Procedure

Amine acceptor **1a**-**k** (0.01 mmol, 10 mM final concentration) was suspended in potassium phosphate buffer (100 mM, pH 7.5). Then, cell-free extract of recombinant ATase (0.066 U) was added to the reaction mixture. The bioacylation was started by addition of the donor at the following final concentrations: DAPG (15 mM), IPEA (100 mM), PA (15 mM). DAPG was dissolved in DMSO (100 µL) to improve its solubility in buffer (10 vol % final concentration). The reaction mixture was shaken for 18 h at 35 ˚C and 750 rpm in an orbital shaker. Reactions were quenched by addition of acetonitrile (1 mL). The precipitated protein was removed by centrifugation (20 min, 14,000 rpm) and the supernatant was subjected to HPLC for determination of conversions. As a negative control, reactions without enzyme were performed.

Semi-preparative-scale Friedel-Crafts bioacetylation of amines

Amine (10 mM final concentration) was dissolved in potassium phosphate buffer (100 mM, pH 7.5) in a shaking flask. Cell-free extract containing the *Pp*ATaseCH (2.5 mL, 1.65 U) was added to the reaction mixture and the bioacetylation was started by adding PA (54.9 µL, 15 mM final concentration). The bioacetylation (25 mL total volume) was run at 35 °C and 140 rpm for 24 h. The resulting suspension was extracted with EtOAc (2 × 50 mL). The organic layers were pooled in a separation funnel, washed with brine (2 × 40 mL), dried over anhydrous Na_2SO_4 and the solvent was removed under reduced pressure. The crude product was purified by flash chromatography using hexane: EtOAc as an eluent. Compounds were characterized by ^{1}H-NMR, ^{13}C-NMR and GC-MS.

Activity Assay

ATase-batch activities were measured on a Thermo Scientific Genesys 10 UV Scanning UV/Vis spectrophotometer according to a modified procedure from literature.[3] When following the disproportionation of MAPG into DAPG and PG spectrophotometrically, an increase of absorption is recorded due to the formation of DAPG (ε = 20 mM^{-1} cm^{-1}, λ = 370 nm). One unit of activity was defined as the µmol of product formed by an enzyme in 1 min per 1 milligram of protein under the following conditions: potassium phosphate buffer (960 µL, 100 mM, pH 7.5) and MAPG (1.2 µmol, 30 µL of a 40 mM stock solution prepared in

DMSO) were added to a cuvette and preheated to 35 °C. The reaction (1 mL total volume, 3 vol% DMSO) was started by the addition of the enzyme-containing cell-free extract (10 µL ≡ 1.43 mg wet cells). The reaction was followed for 1 minute. All reactions were performed as a duplicate. The protein concentration (Bradford) was measured [(ε = 0.083 mL mg^{-1} cm^{-1}, λ = 595 nm)] and specific activities were determined as units per mg protein.

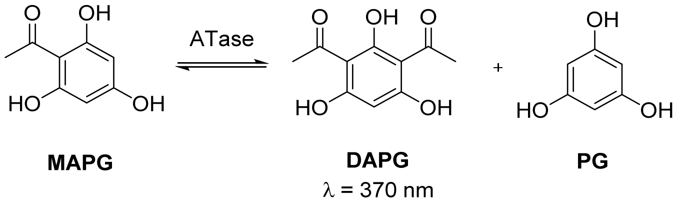

Scheme S1. Reaction scheme of assay.

Purification of *Pp*ATaseCH

Purification of the *Pp*ATaseCH was achieved by size-exclusion chromatography with a Superdex 200 16/600 HiLoad-column. The column was initially washed with water, followed by conditioning (potassium phosphate buffer, 50 mM, pH 7.5, 100 mM NaCl). The cell-free extract (4 mL ≡ 0.57 g wet cells) was filtered (0.45 µm) prior to loading onto the column. The PpATaseCH eluted after ~66 min with a flow-rate of 0.75 mL min^{-1} and the size of the protein (~98 kDa) was determined by comparison to a GelFiltration standard (BioRad). The purity of the *Pp*ATaseCH-fractions was estimated by SDS-PAGE (Figure S1). All enzyme-containing fractions (10 × 500 µL) were combined and concentrated to approximately 2.5 mL with a Vivaspin column (MWCO 30,000). NaCl was removed by filtration through a PD-10-desalting column (final buffer = potassium phosphate buffer, 50 mM, pH 7.5) and the enzyme solution was concentrated again. Initial rates and protein concentrations were measured to determine the batch activity. In total, 13.1 mg of purified enzyme (0.1 U mg^{-1}) was obtained from 0.57 g wet cells.

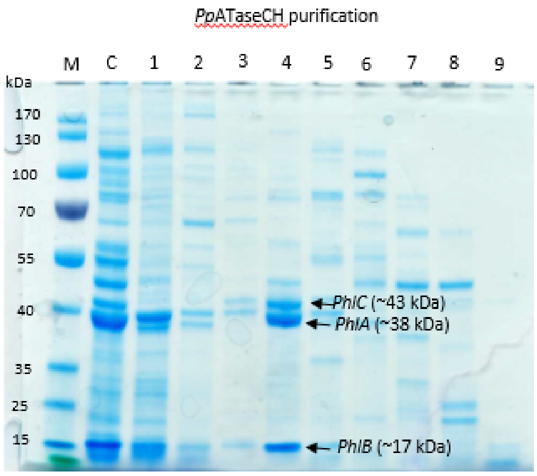

Figure S1. SDS-PAGE analysis of the *Pp*ATaseCH after purification *via* size-exclusion chromatography. Cell-free extract (M), flow-through (lanes 1-3; 5-9) and purified fractions of *Pp*ATaseCH (lane 4).

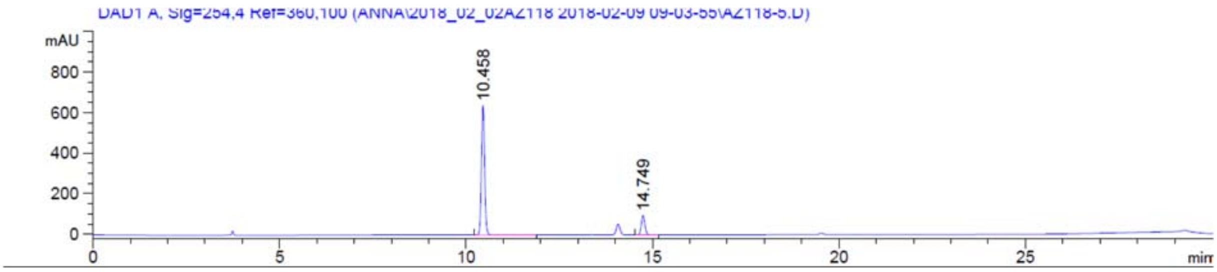

Figure S2. HPLC-chromatogram showing analytical-scale *N*-acylation of **1a** into **2a** (t_r = 10.5 min) using PA (t_r = 14.8 min) as an acyl donor and purified ATase as a catalyst (conversion >99%).

Chemical Synthesis - General Procedure for Synthesis of Reference Compounds

In a round-bottomed flask, a mixture of a suitable amine (1 mmol) and vinyl acetate (1 mL, 9.2 mmol) was stirred at room temperature for 24 h. The progression of the reaction was monitored by TLC. Crude reaction mixture was concentrated in vacuo and purified by column chromatography on silica gel (hexane/ethyl acetate) to afford corresponding acetamides.[2]

_N_-(3-hydroxyphenyl)acetamide (**2a**)

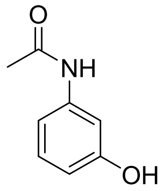

N-(3-hydroxyphenyl)acetamide was chemically obtained as a white solid with 88 % product yield. ^{1}H-NMR (300 MHz, acetone-*d6*): δ [ppm] = 2.07 (s, 3H), 6.53 (dd, J_1 = 1.9 Hz, J_2=8.5 Hz, 1H), 6.97 (dd, J_1 = 3.9 Hz, J_2=4.9 Hz, 1H), 7.09 (t, *J* = 8.0 Hz, 1H, Ar), 7.41 (t, *J* = 2.1 Hz, 1H), 8.40 (s, 1H, OH), 9.1 (s, 1H, NH); ^{13}C-NMR (75 MHz, acetone-*d6*): δ_C [ppm] = 23.4, 106.3, 110.2, 129.3, 140.8, 157.9, 168.1.

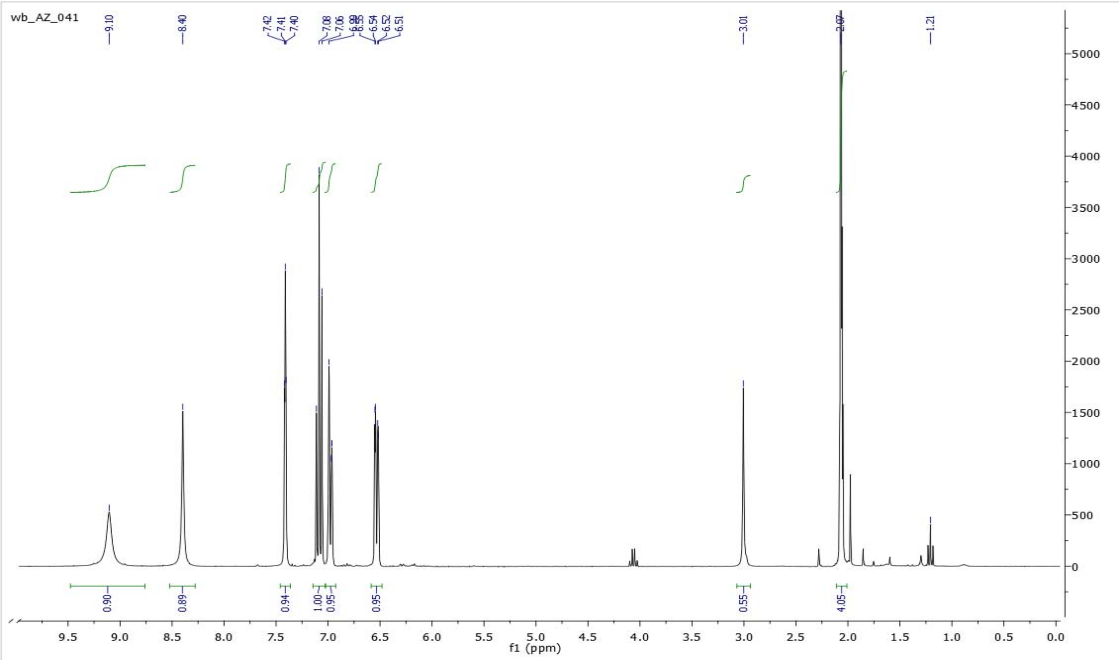

Figure S3. ^{1}H-NMR of compound **2a.**

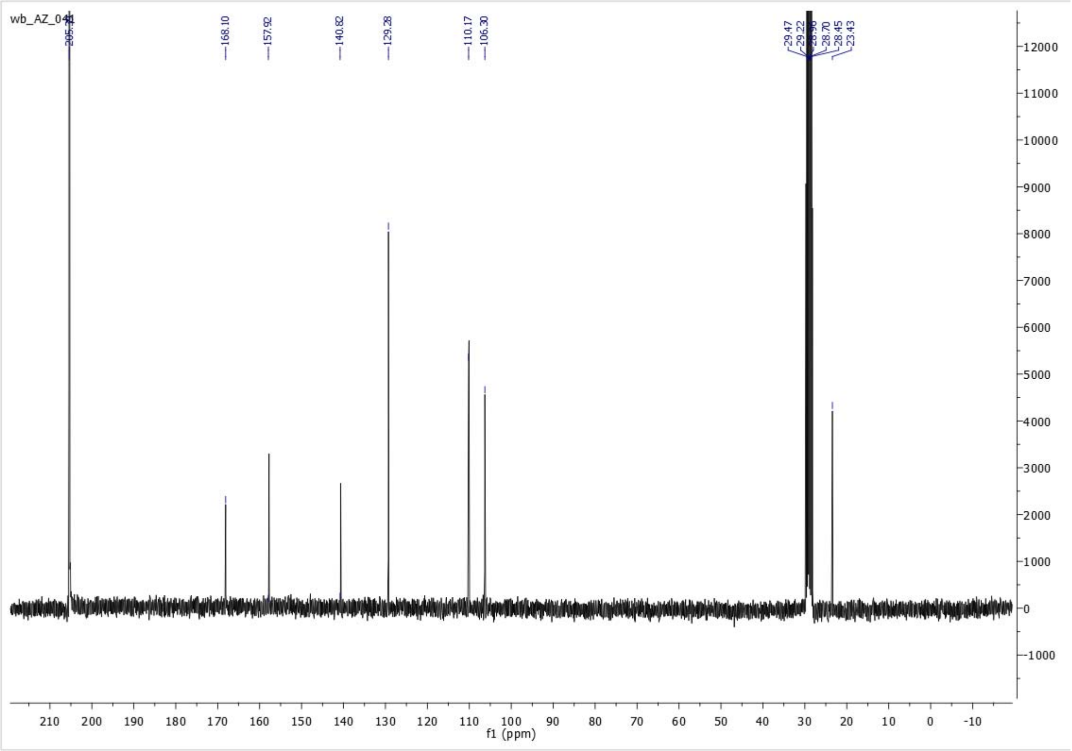

Figure S4. ^{13}C-NMR of compound **2a.**

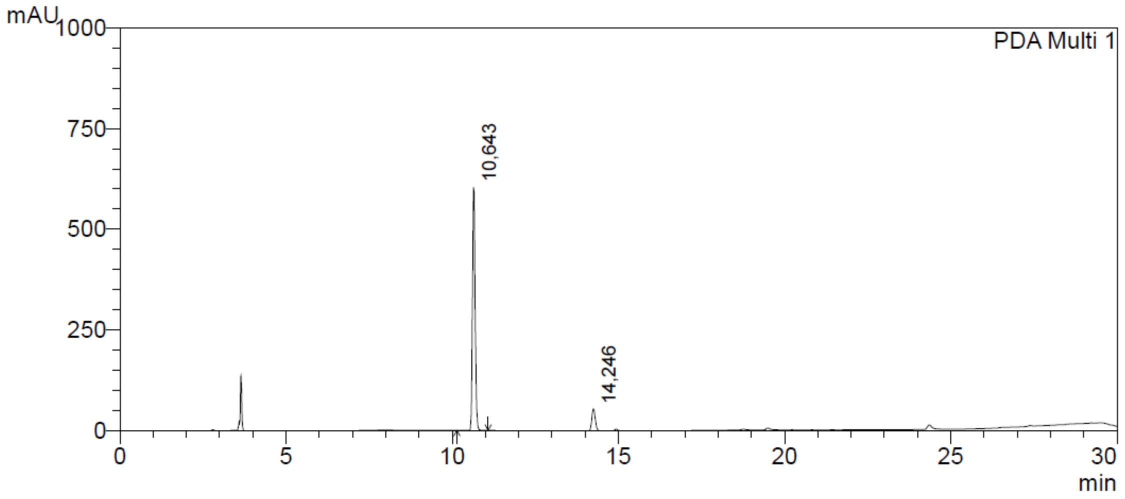

Figure S5. HPLC-chromatogram showing analytical-scale *N*-acetylation of **1a** into **2a** (t_r = 10.6 min) using PA (t_r = 14.2 min) as an acetyl donor.

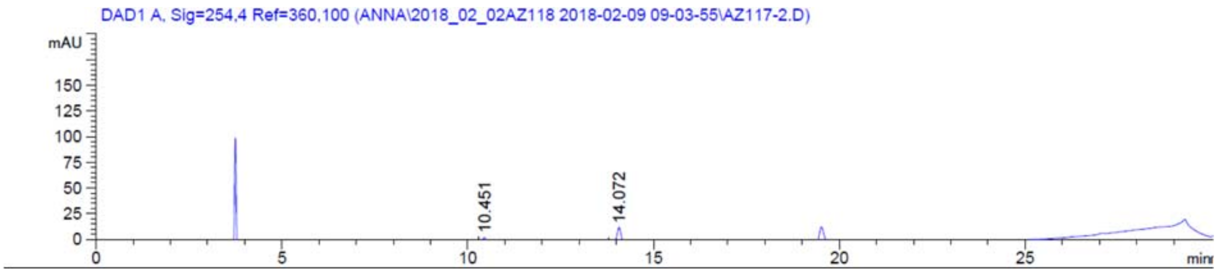

Figure S6. HPLC-chromatogram showing the analytical-scale *N*-acylation of **1a** into **2a** (t_r = 10.5 min) using PA (t_r = 14.1 min) as an acyl donor and *E. coli* free-extract containing no acyltransferase (conversion ~1%).

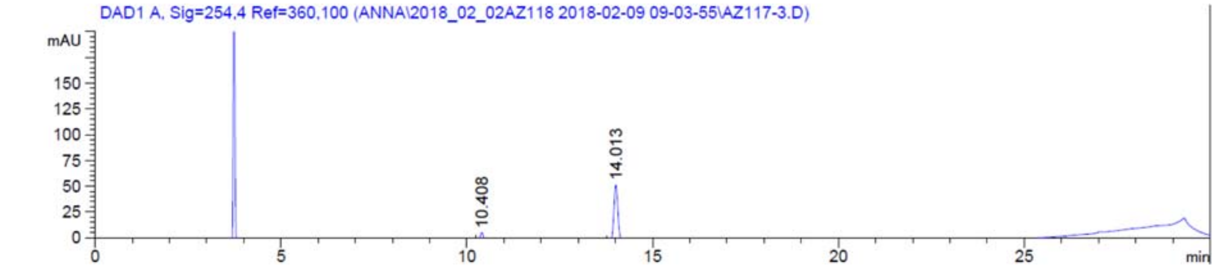

Figure S7. HPLC-chromatogram showing analytical-scale *N*-acylation of **1a** into **2a** (t_r = 10.4 min) using PA (t_r = 14.0 min) as an acyl donor in the absence of any catalyst (conversion ~1%, blank reaction).

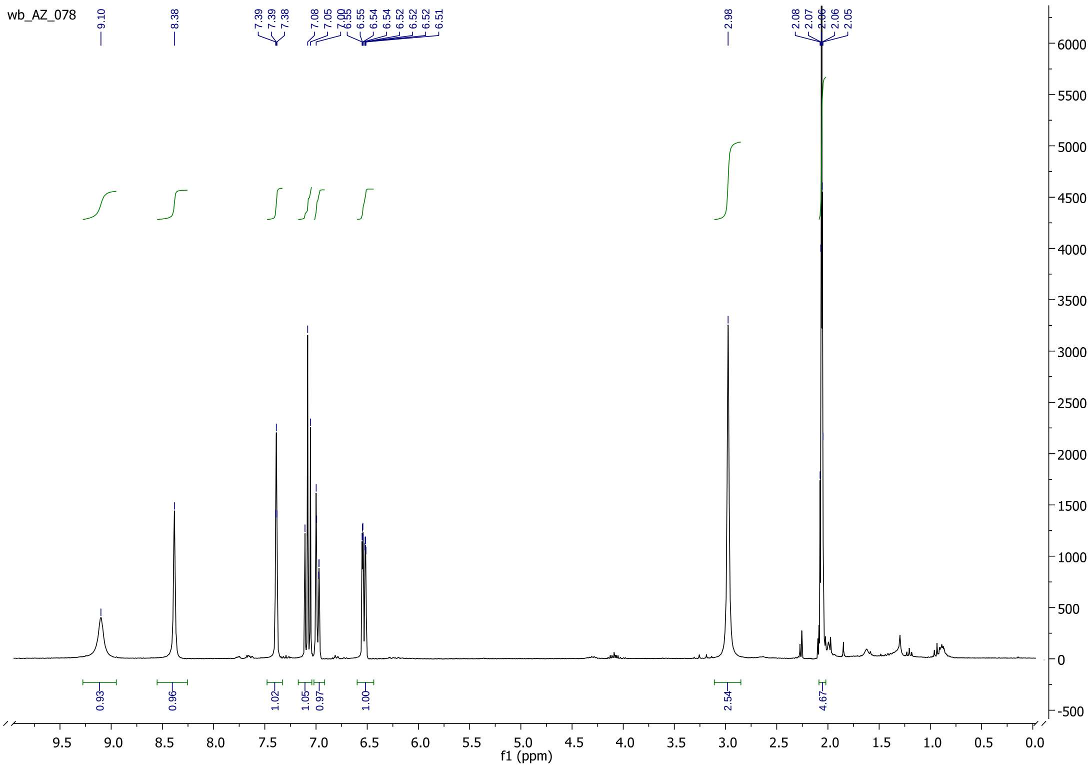

Figure S8. ^{1}H-NMR of compound **2a** isolated from the preparative scale bioacylation.

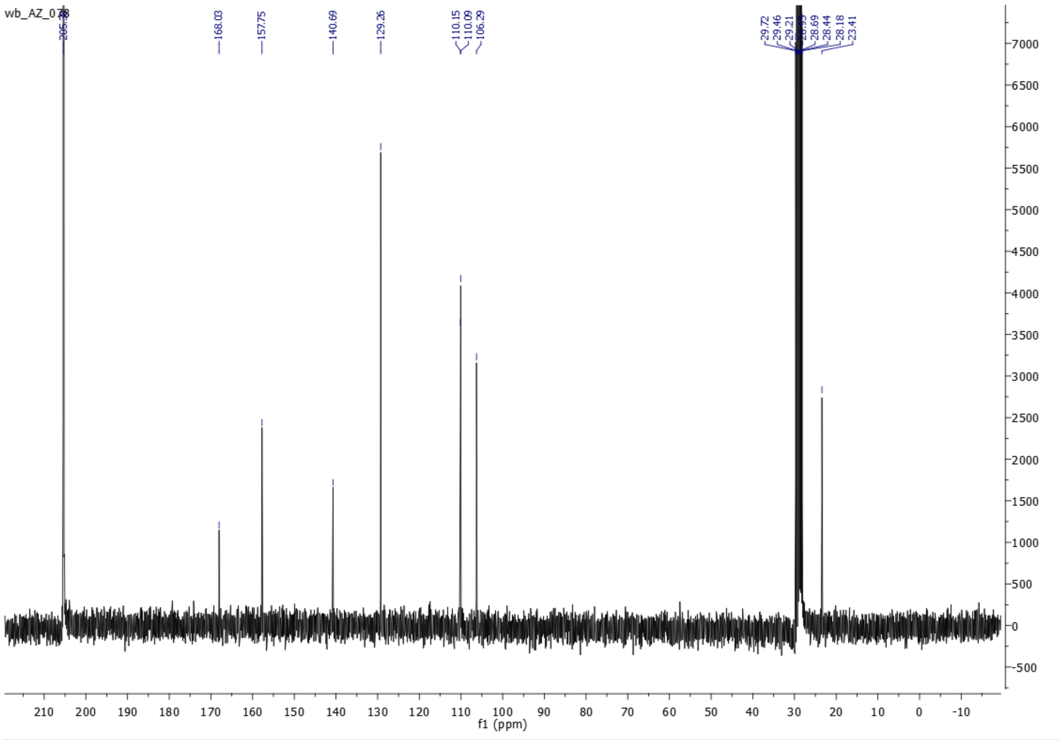

Figure S9. ^{13}C-NMR of compound **2a** isolated from the preparative scale bioacylation.

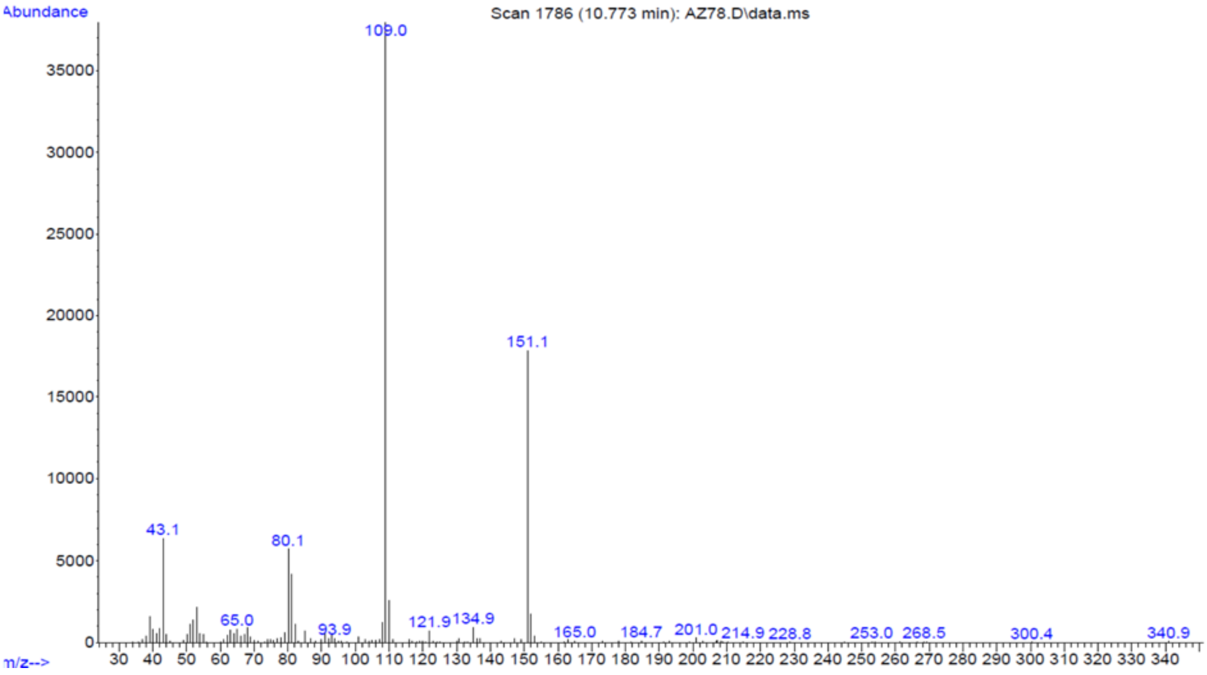

Figure S10. MS of compound **2a** isolated from the preparative scale bioacylation.

N-phenylacetamide (2e)

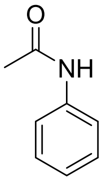

N-phenylacetamide was purchased from Sigma-Aldrich. *N*-phenylacetamide isolated after biotransformation: ^{1}H-NMR (300 MHz, $CDCl_3$): δ [ppm] = 2.18 (s, 3H), 7.12 (t, *J* = 7.4 Hz, 1H), 7.31 (dd, J_1 = 14.4 Hz, J_2 = 6.2 Hz, 2H), 7.52 (d, *J* = 7.7 Hz, 2H), 7.60 (s, 1H); ^{13}C-NMR (75 MHz, $CDCl_3$): δ_C [ppm] = 24.6, 120.0, 124.3, 129.0, 137.9, 168.6.

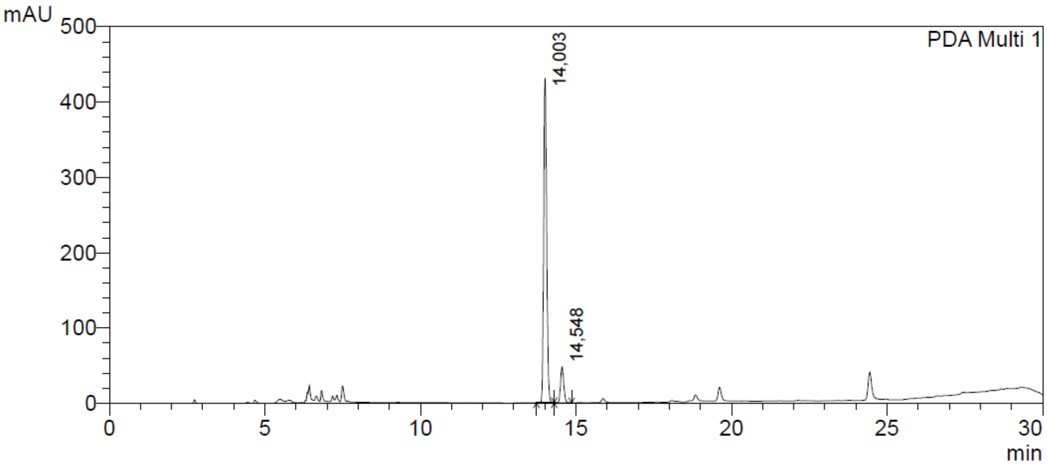

Figure S11. HPLC-chromatogram showing analytical-scale *N*-acylation of **1e** into **2e** (t_r = 14.0 min) using PA (t_r = 14.5 min) as an acyl donor.

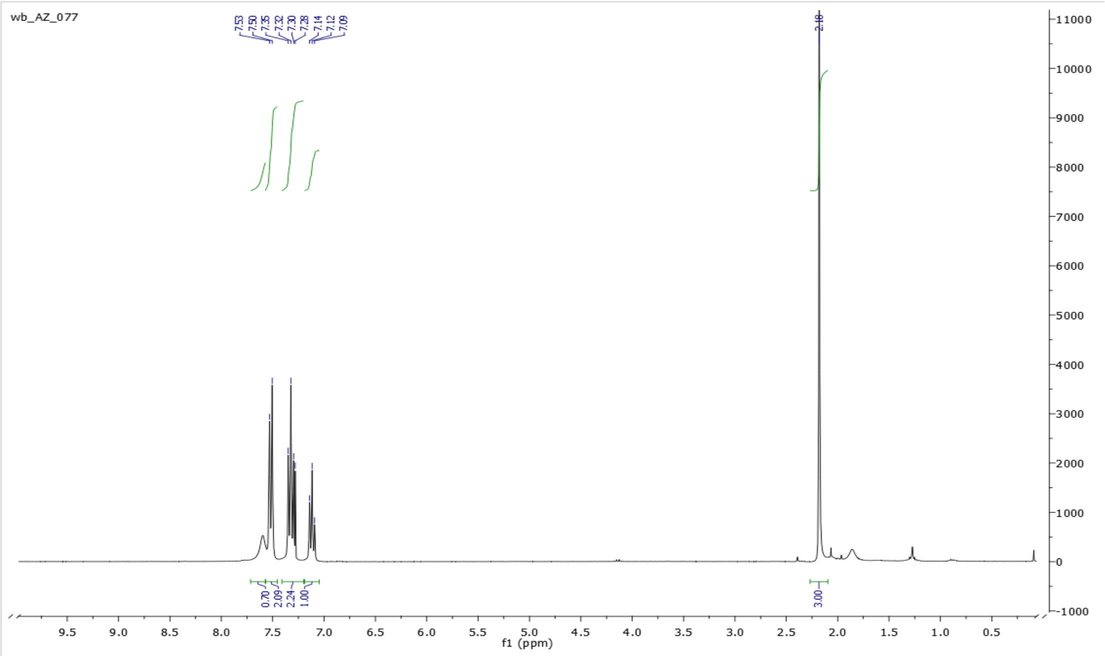

Figure S12. ^{1}H-NMR of compound **2e** isolated from the preparative scale bioacylation.

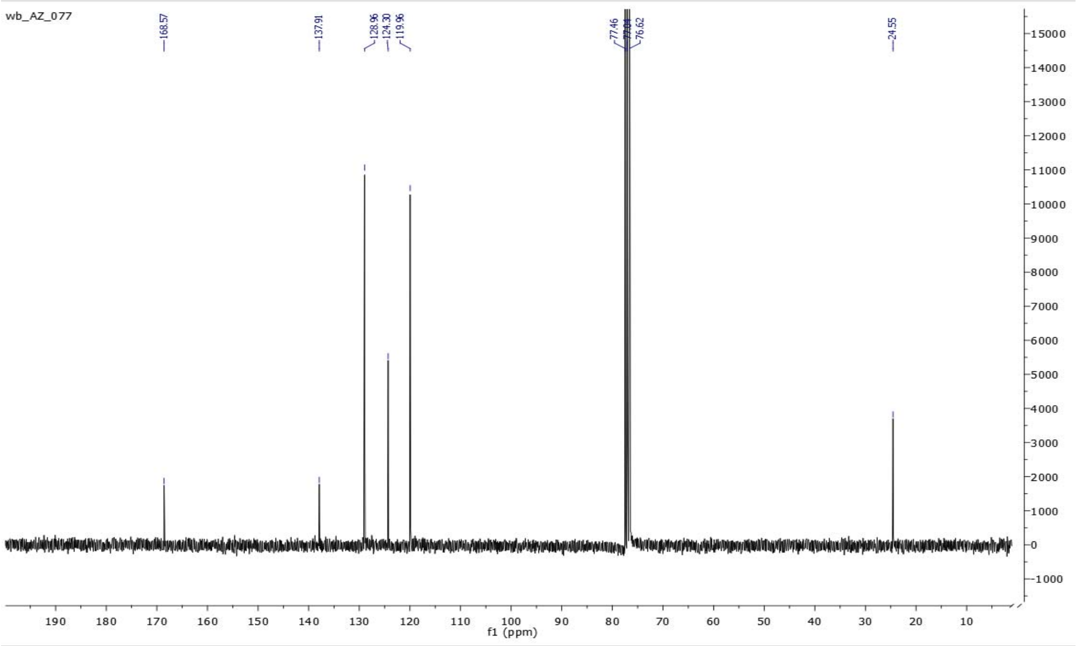

Figure S13. ^{13}C-NMR of compound **2e** isolated from the preparative scale bioacylation.

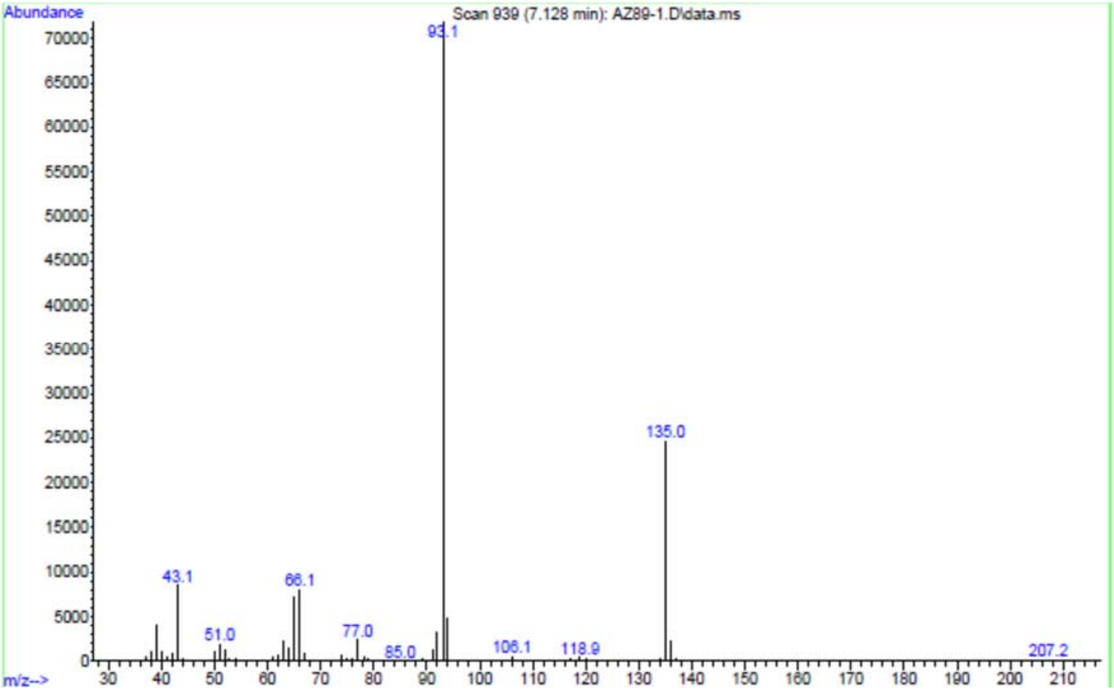

Figure S14. MS of compound **2e** isolated from the preparative scale bioacylation.

N-(4-chlorophenyl)acetamide (2f)

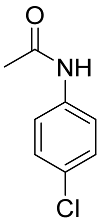

N-(4-chlorophenyl)acetamide was chemically obtained as a white solid with 28 % product yield. ^{1}H-NMR (300 MHz, acetone-*d6*): δ [ppm] = 2.09 (s, 3H), 7.31 (d, *J* = 8.9 Hz, 2H, Ar), 7.68 (d, *J* = 9.9 Hz, 2H), 9.29 (s, 1H, NH). ^{13}C-NMR (75 MHz, acetone-*d6*): δ_C [ppm] = 23.3, 120.4, 127.3, 128.5, 138.5, 168.1.

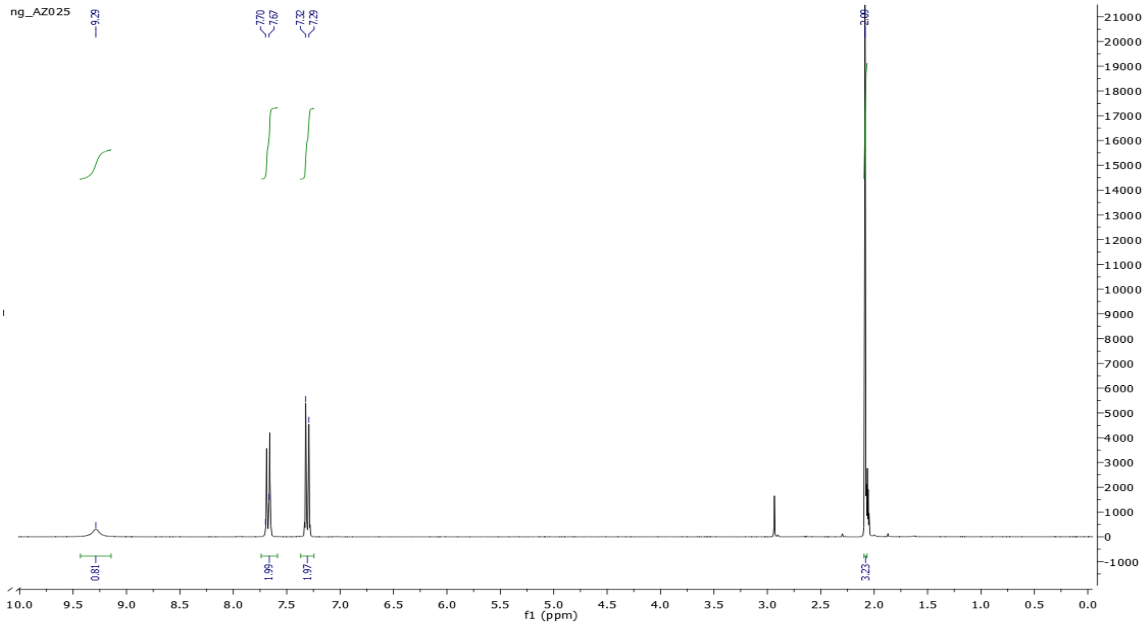

Figure S15. ^{1}H-NMR of compound **2f.**

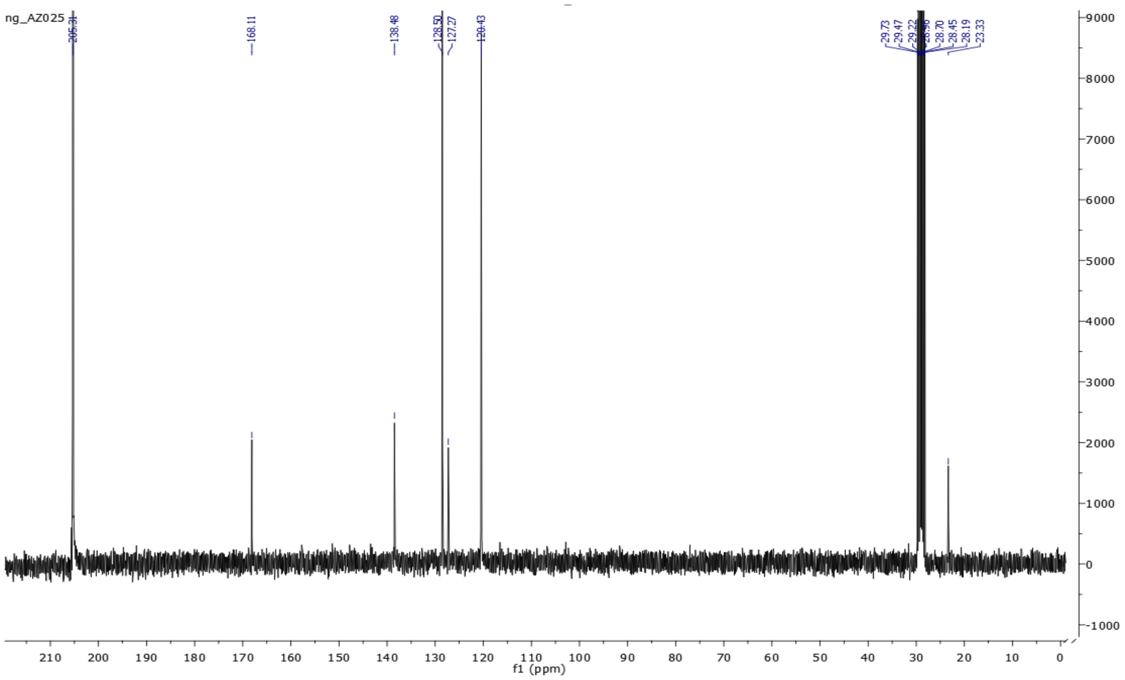

Figure S16. ^{13}C-NMR of compound **2f.**

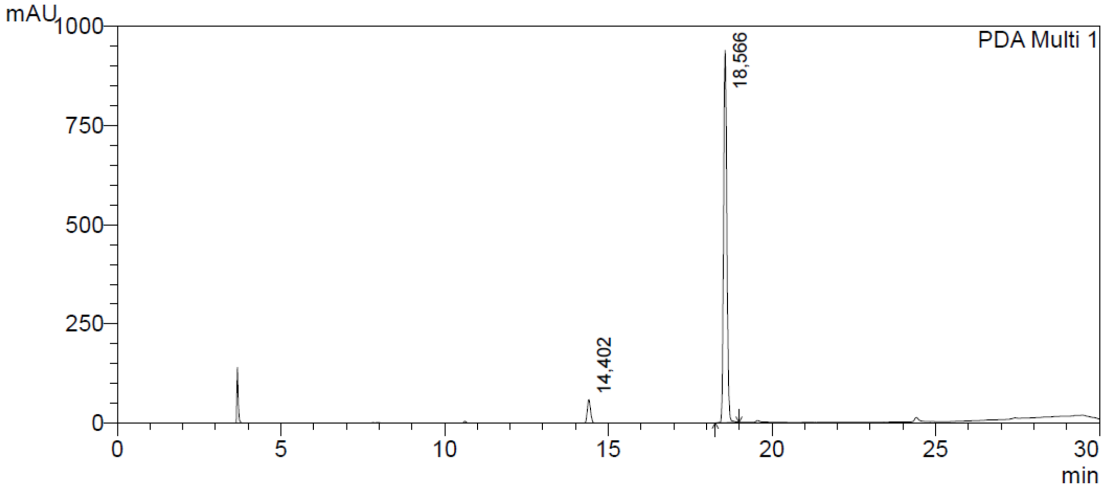

Figure S17. HPLC-chromatogram showing analytical-scale *N*-acylation of **1f** into **2f** (t_r = 18.6 min) using PA (t_r = 14.4 min) as an acyl donor.

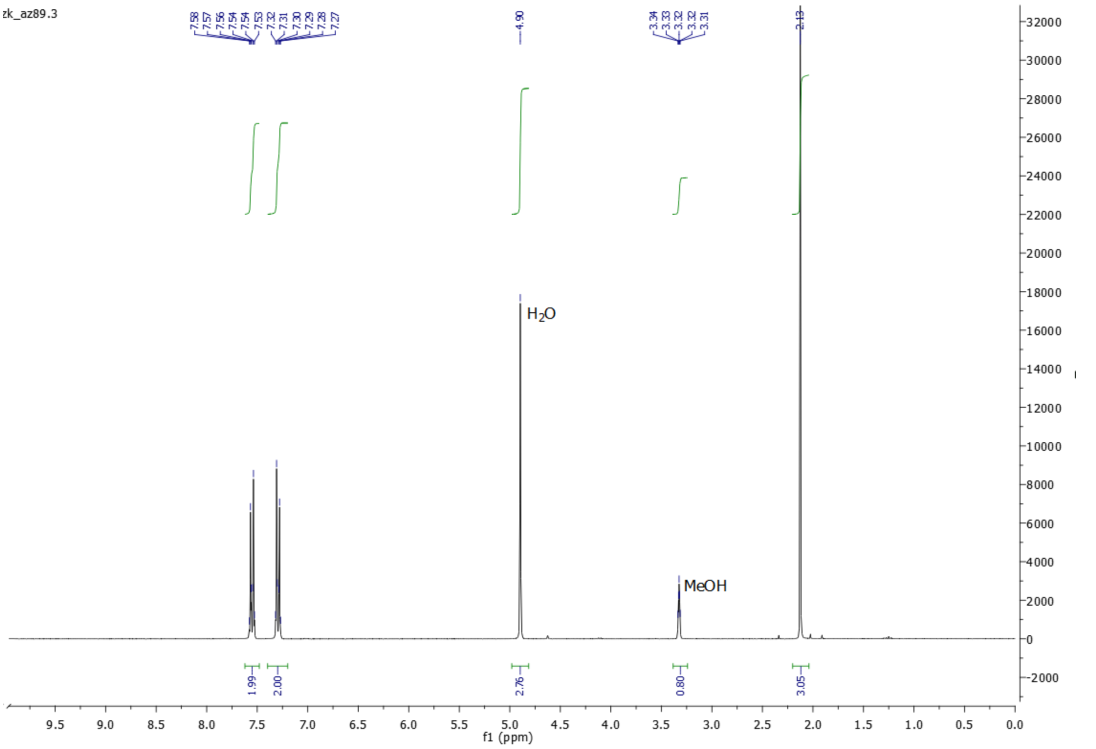

Figure S18. ^{1}H-NMR of compound **2f** isolated from the preparative scale bioacylation.

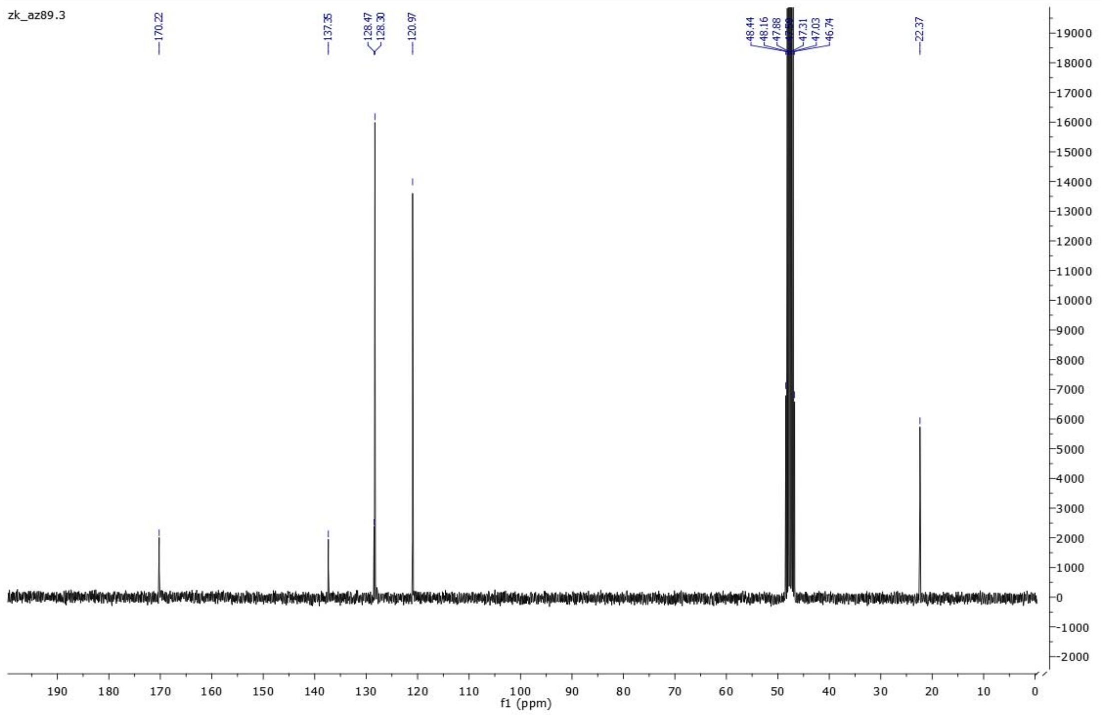

Figure S19. ^{13}C-NMR of compound **2f** isolated from the preparative scale bioacylation.

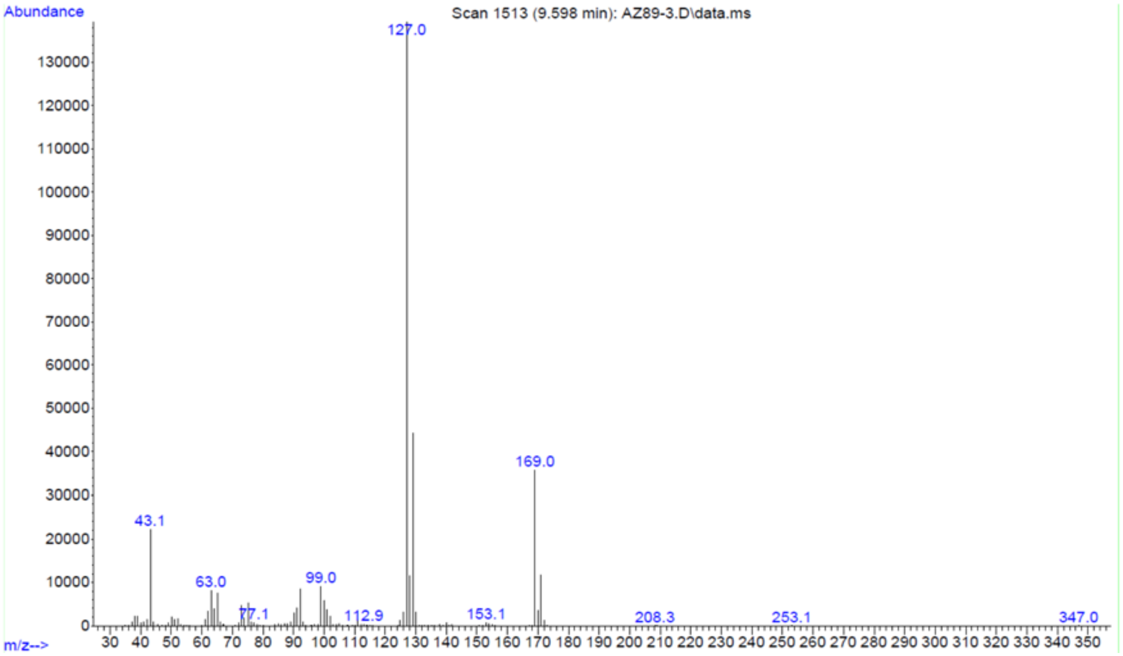

Figure S20. MS of compound **2f** isolated from the preparative scale bioacylation.

***N*-(4-isopropylphenyl)acetamide** (**2g**)

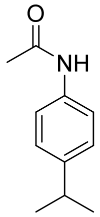

N-(4-isopropylphenyl)acetamide was chemically obtained as a white solid with 95 % product yield. ^{1}H-NMR (300 MHz, acetone-*d6*): δ [ppm] = 1.21 (d, *J* = 6.9 Hz, 6H), 2.06 (s, 3H), 2.86 (dt, J_1 = 6.8 Hz, J_2 = 13.8 Hz, 1H), 7.07-7.24 (m, 2H), 7.46-7.62 (m, 2H), 9.08 (s, 1H, N<u>H</u>). ^{13}C-NMR (75 MHz, acetone-*d6*): δ_C [ppm] = 23.5, 23.5, 33.4, 119.1, 126.3, 137.4, 143.5, 167.7.

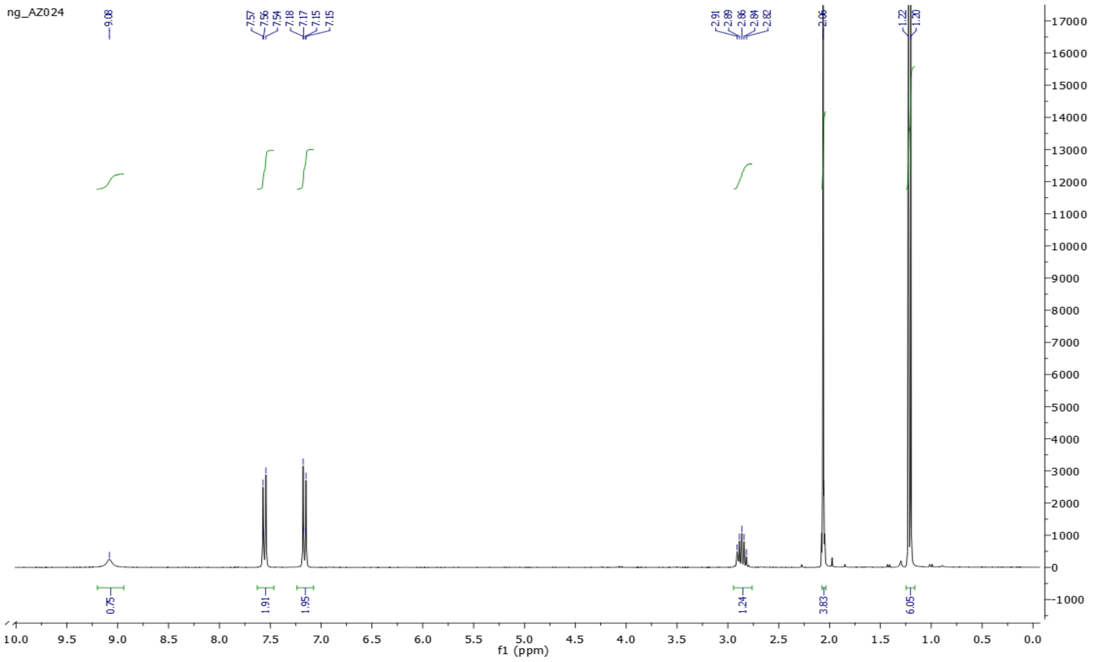

Figure S21. ^{1}H-NMR of compound **2g.**

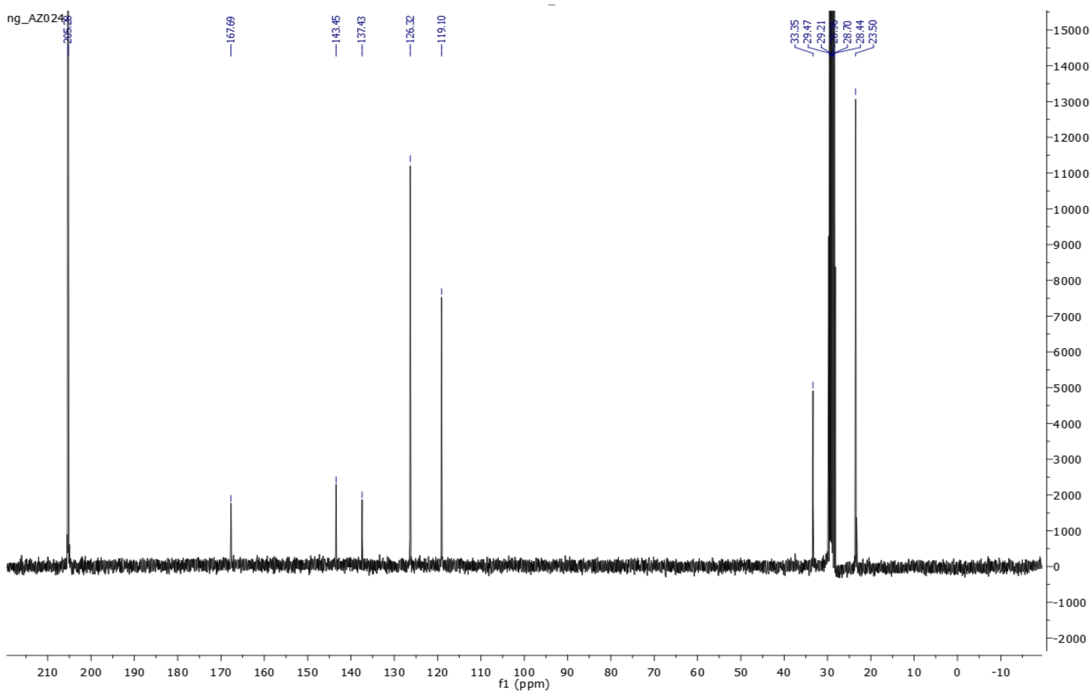

Figure S22. ^{13}C-NMR of compound **2g.**

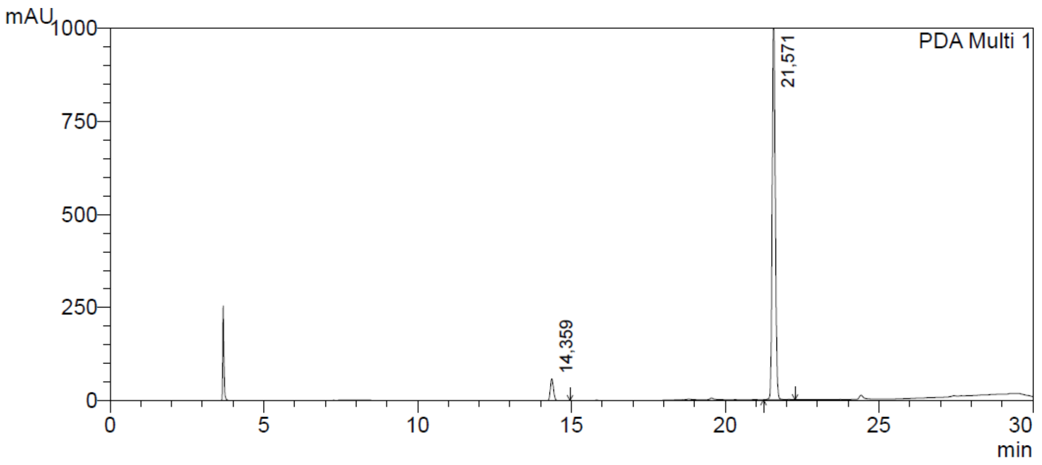

Figure S23. HPLC-chromatogram showing analytical-scale *N*-acylation of **1g** into **2g** (t_r = 21.6 min) using PA (t_r = 14.4 min) as an acyl donor.

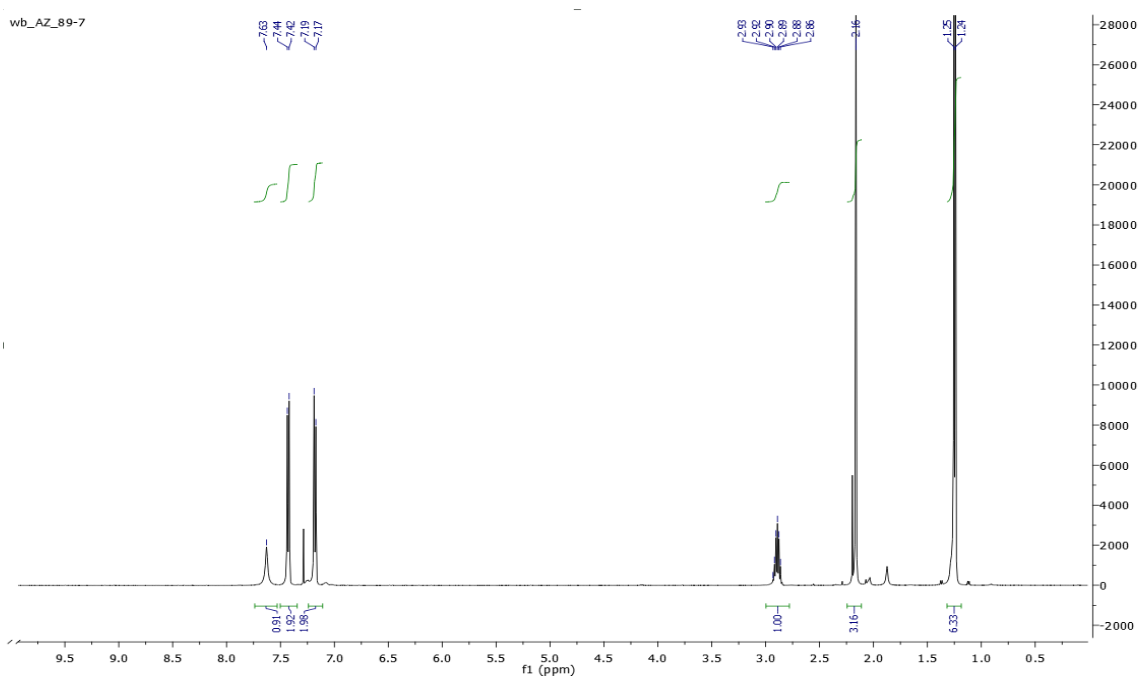

Figure S24. ^{1}H-NMR of compound **2g** isolated from the preparative scale bioacylation.

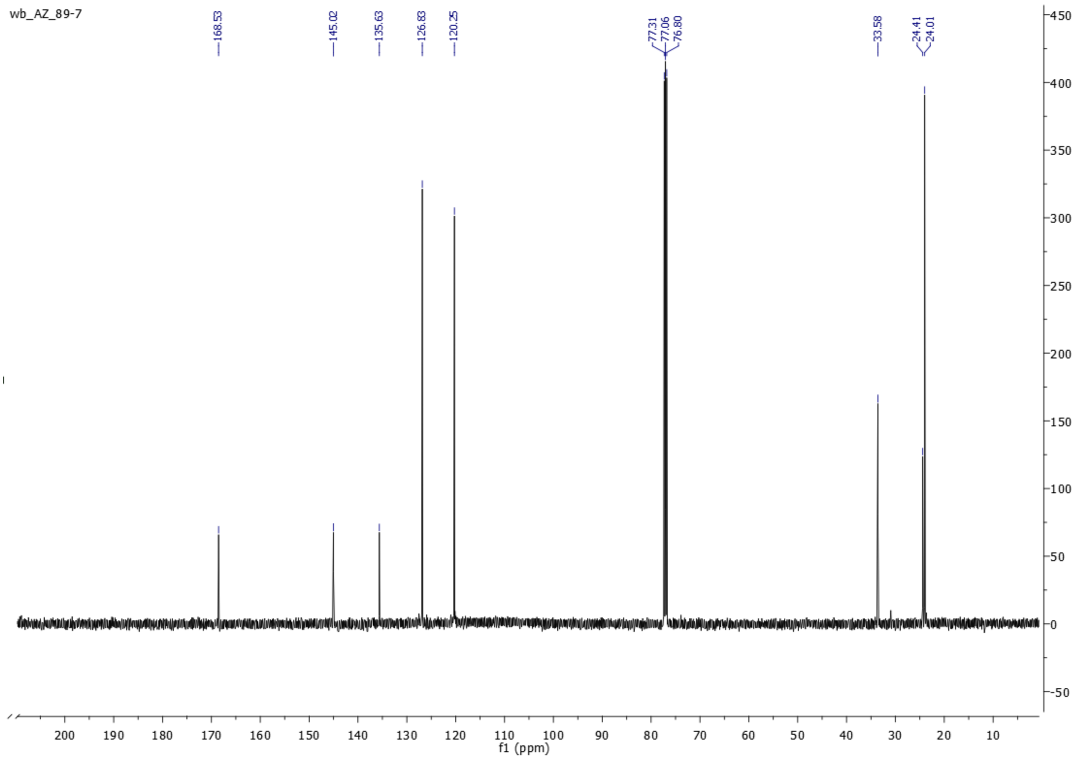

Figure S25. ^{13}C-NMR of compound **2g** isolated from the preparative scale bioacylation.

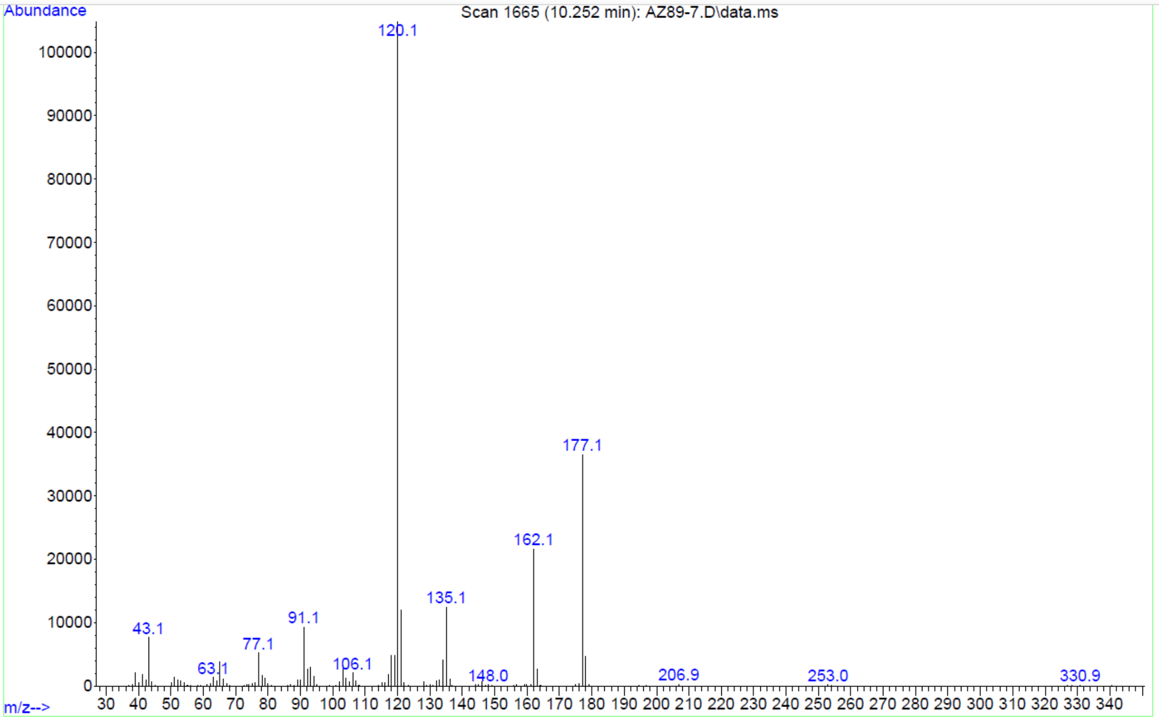

Figure S26. MS of compound **2g** isolated from the preparative scale bioacylation.

N-(4-ethylphenyl)acetamide (**2h**)

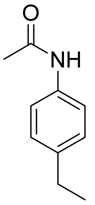

N-(4-ethylphenyl)acetamide was chemically obtained as a pale yellow solid with 40 % product yield. ^{1}H-NMR (300 MHz, acetone-*d6*): δ [ppm] = 1.19 (t, *J* = 7.6 Hz, 3 H), 2.06 (s, 3H), 2.58 (q, *J* = 7.6 Hz, 2H). 7.13 (d, *J* = 8.5 Hz, 2H), 7.55 (d, *J* = 8.5 Hz, 2H), 9.08 (s , 1H, N<u>H</u>), ^{13}C-NMR (75 MHz, acetone-*d6*): δ_C [ppm] = 14.9, 23.3, 28.4, 119.1, 127.8, 137.4 (2 x arom. C), 138.9 (2 × arom. C), 167.6.

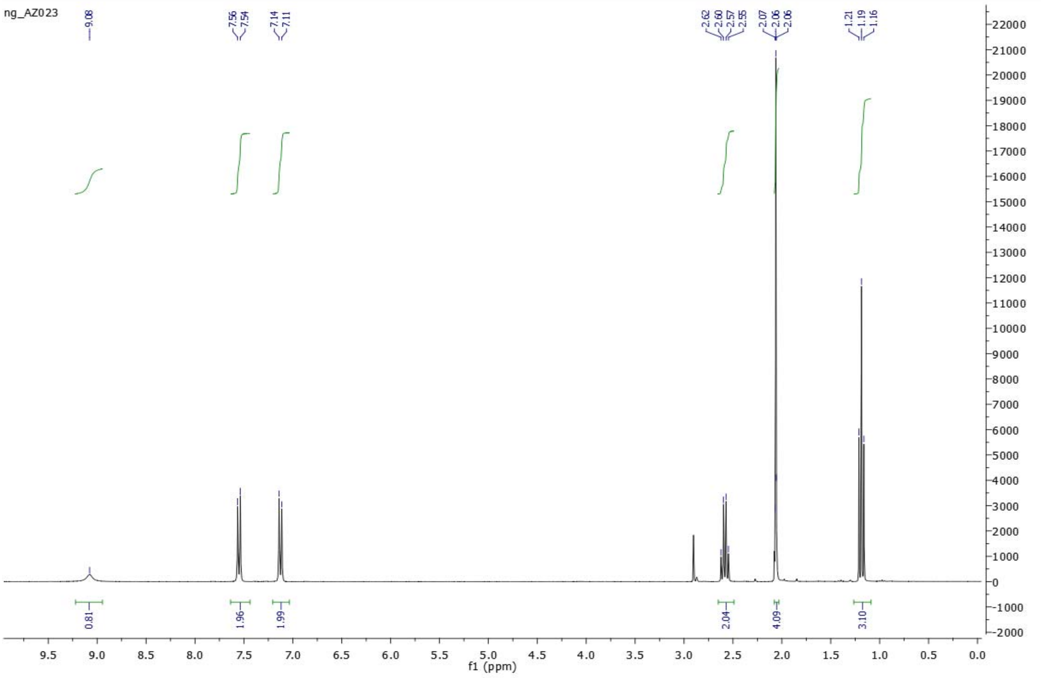

Figure S27. ^{1}H-NMR of compound **2h**

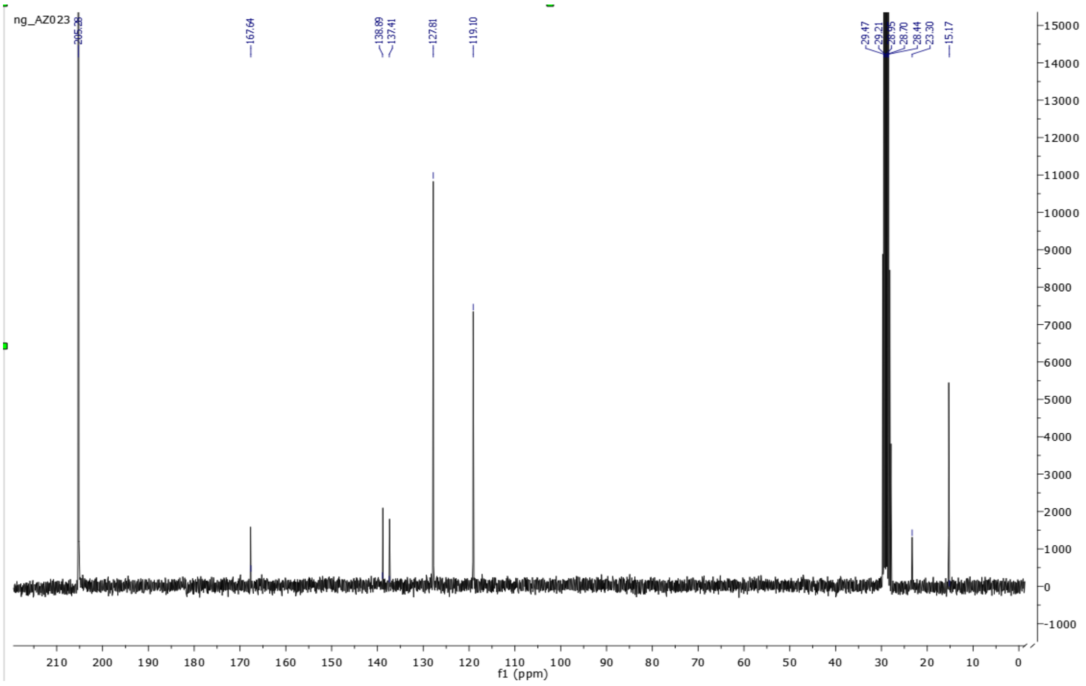

Figure S28. ^{13}C-NMR of compound **2h.**

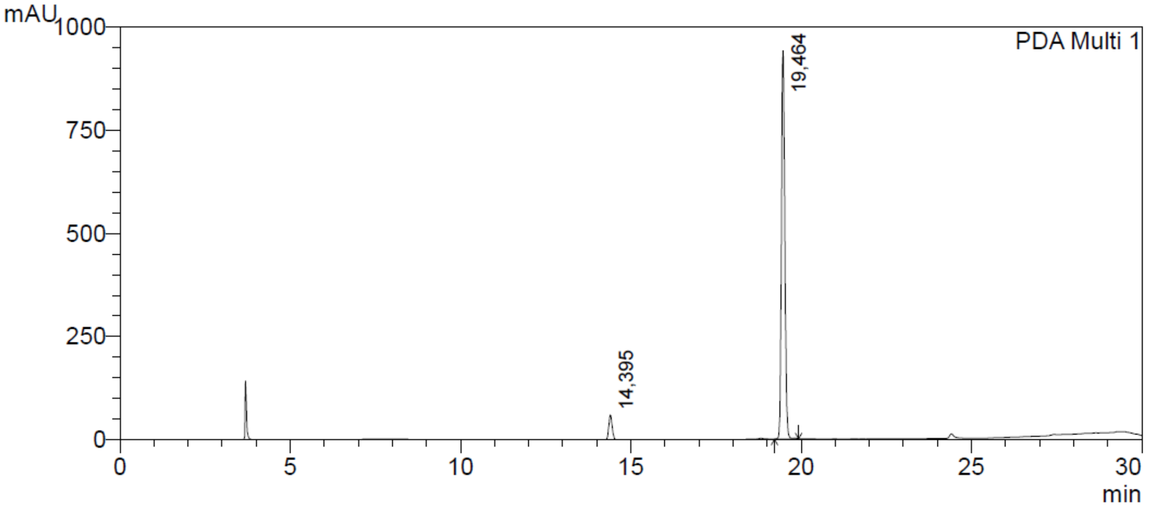

Figure S29. HPLC-chromatogram showing analytical-scale *N*-acylation of **1h** into **2h** (t_r = 19.4 min) using PA (t_r = 14.4 min) as an acyl donor.

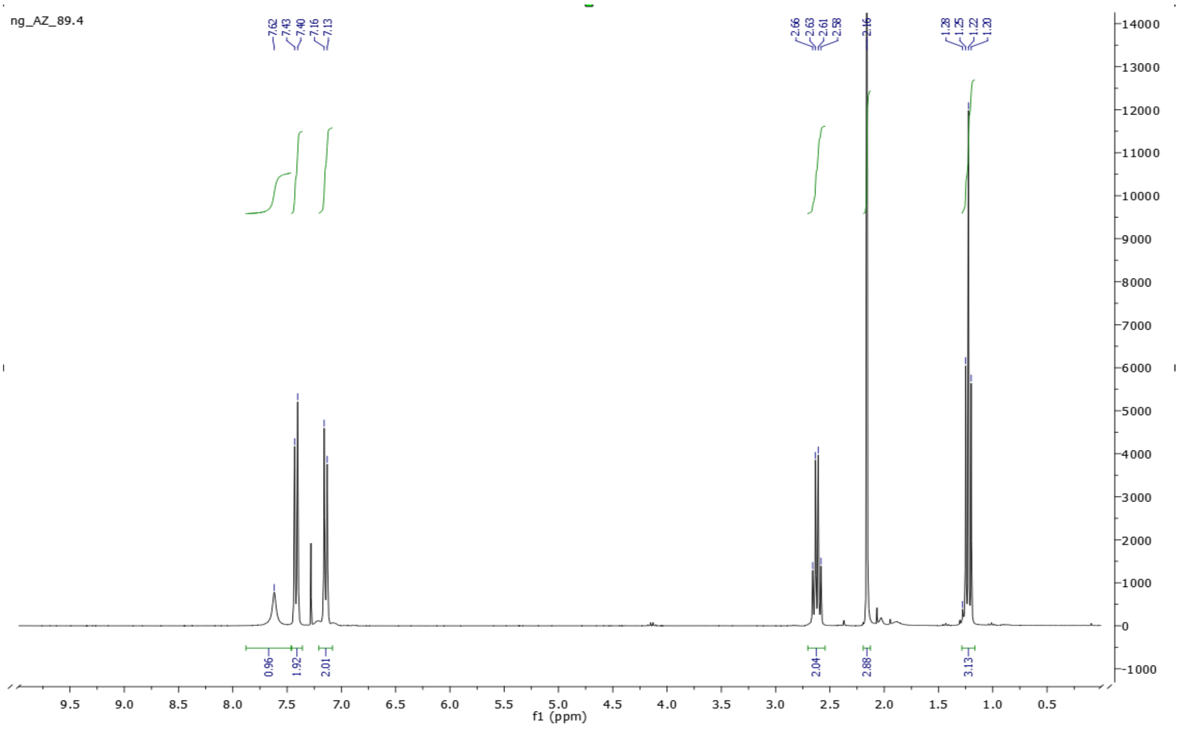

Figure S30. ^{1}H-NMR of compound **2h** isolated from the preparative scale bioacylation.

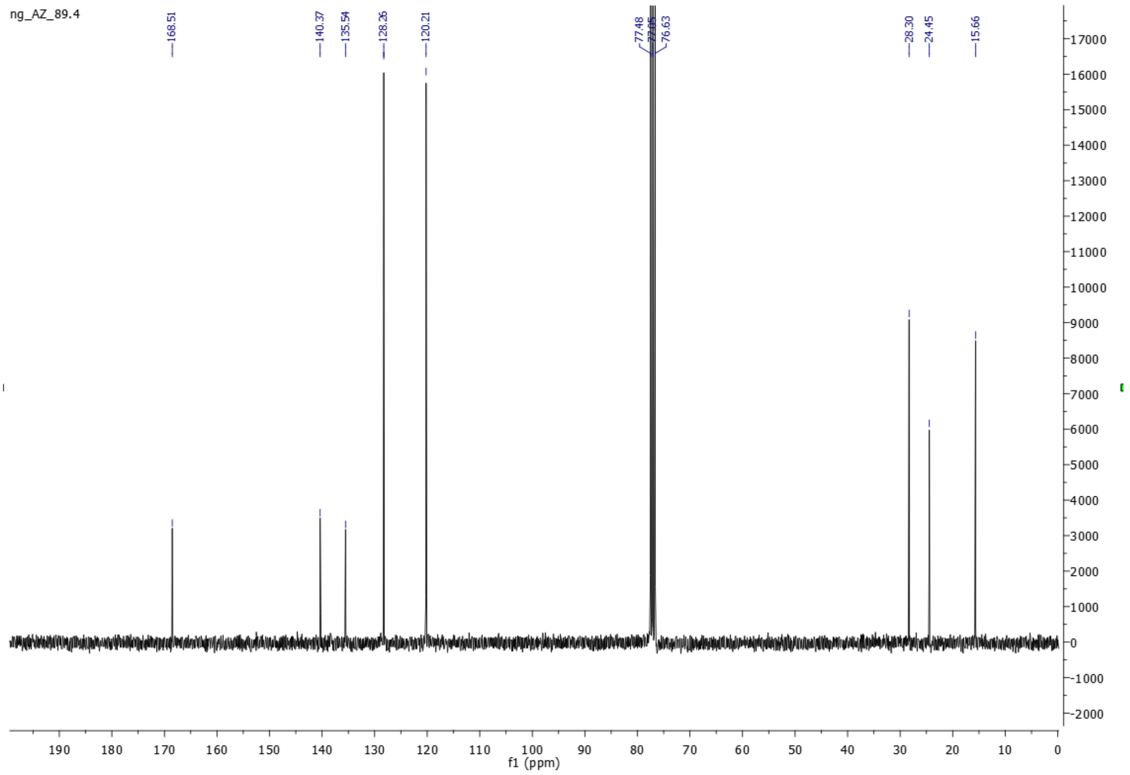

Figure S31. ^{13}C-NMR of compound **2h** isolated from the preparative scale bioacylation.

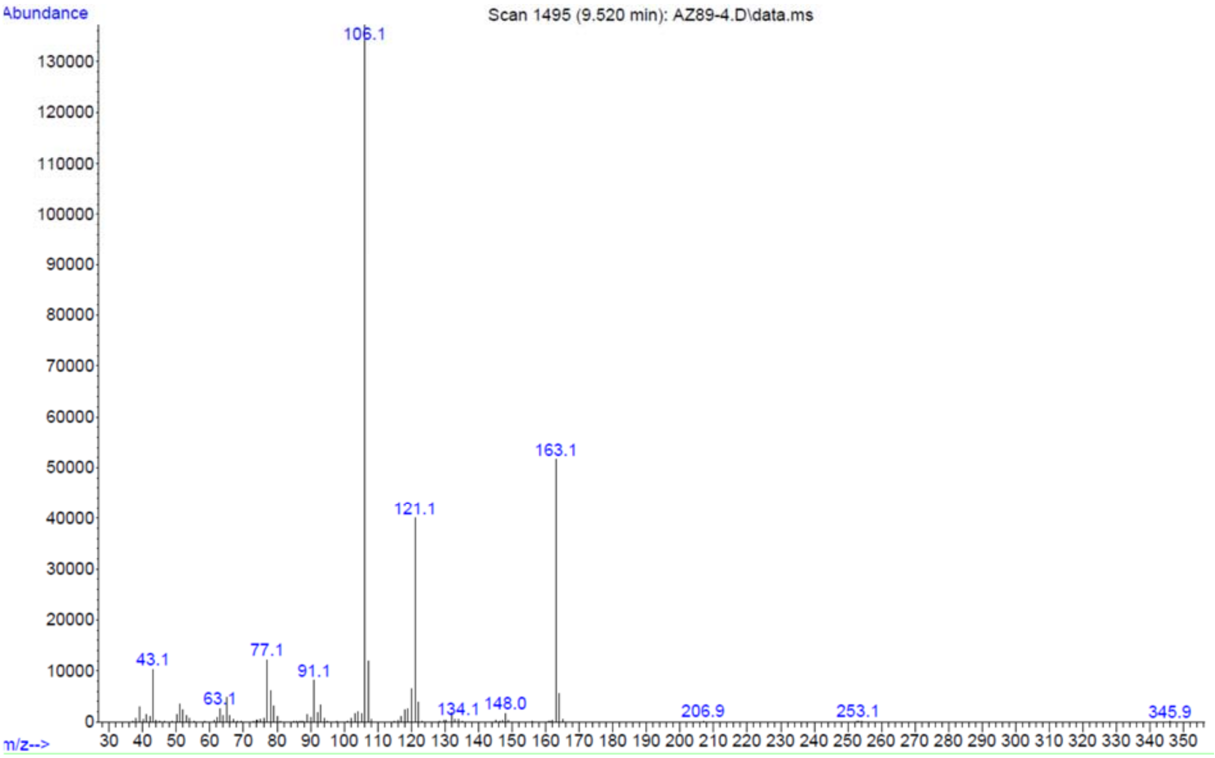

Figure S32. MS of compound **2h** isolated from the preparative scale bioacylation.

N-(3-ethylphenyl)acetamide (2i)

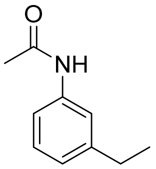

N-(3-ethylphenyl)acetamide was chemically obtained as a white solid with 67 % product yield. ^{1}H-NMR (300 MHz, $CDCl_3$): δ [ppm] = 1.23 (t, *J* = 7.6 Hz, 3 H), 2.17 (s, 3H), 2.63 (q, *J* = 7.6 Hz, 2H). 6.96 (d, *J* = 7.5 Hz, 1H), 7.15-7.42 (m, 3H), 7.60 (s, 1H, N<u>H</u>); ^{13}C-NMR (75 MHz, $CDCl_3$): δ_C [ppm] = 15.5, 24.6, 28.8, 117.3, 119.5, 123.9, 128.8, 137.9, 145.2, 168.5.

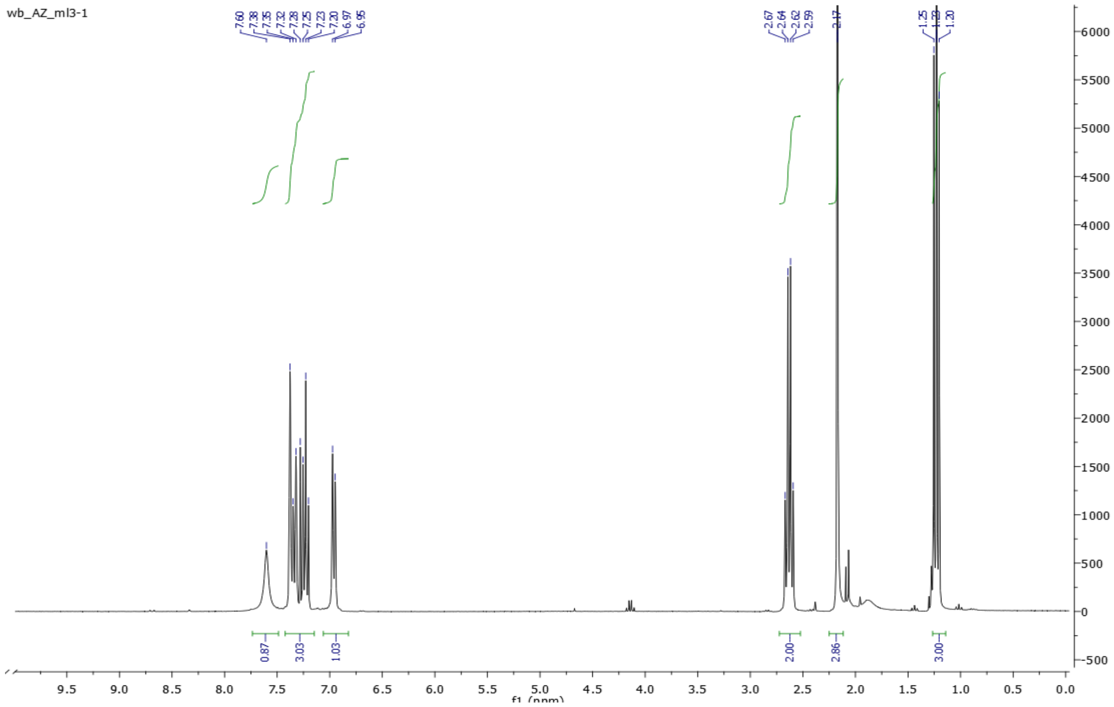

Figure S33. ^{1}H-NMR of compound **2i**.

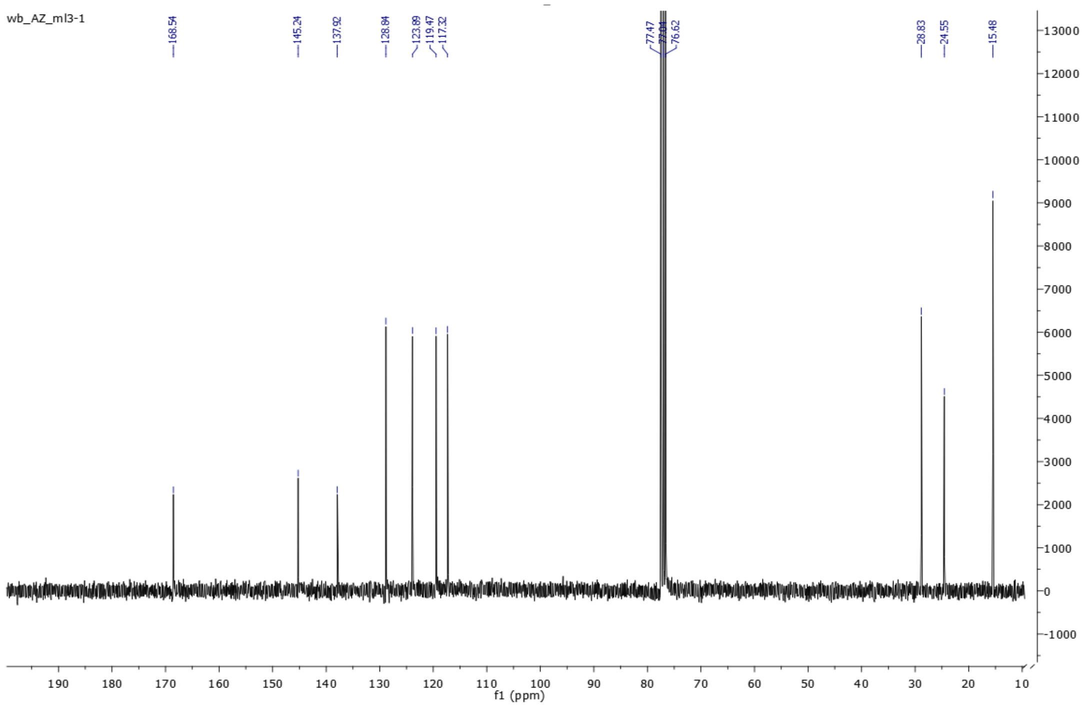

Figure S34. ^{13}C-NMR of compound **2i**.

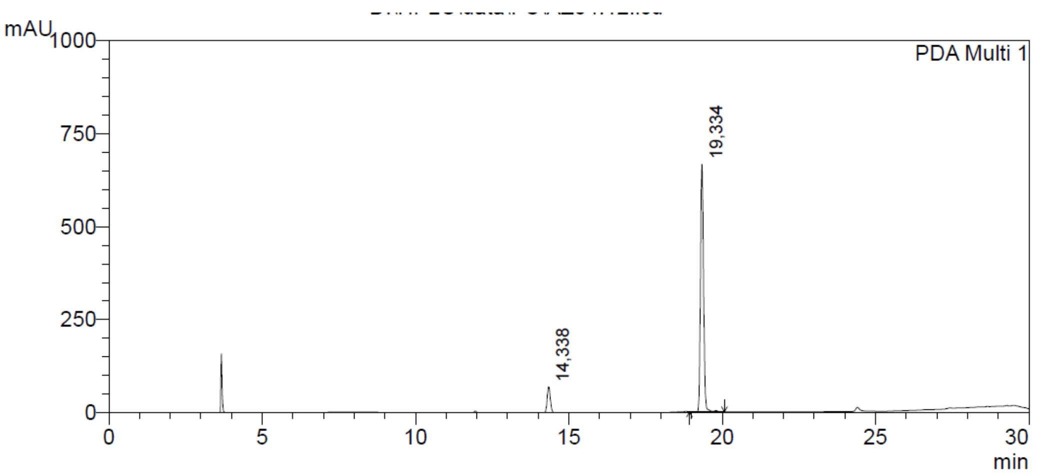

Figure S35. HPLC-chromatogram showing analytical-scale *N*-acylation of **1i** into **2i** (t_r = 19.3 min) using PA (t_r = 14.3 min) as an acyl donor.

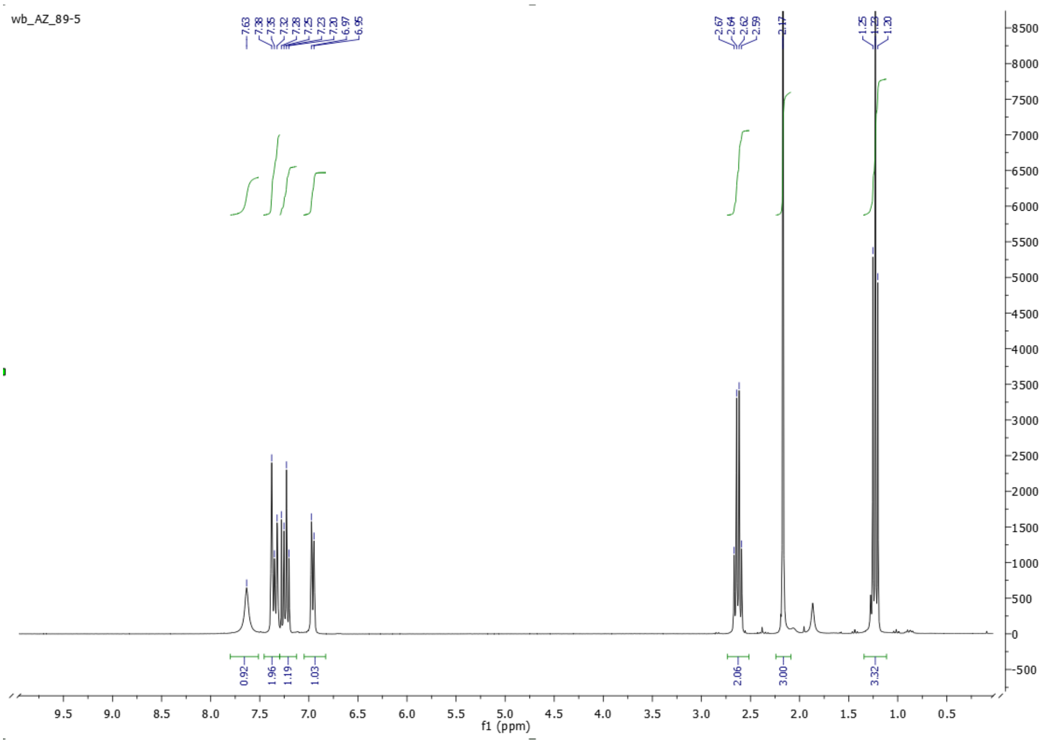

Figure S36. ^{1}H-NMR of compound **2i** isolated from the preparative scale bioacylation.

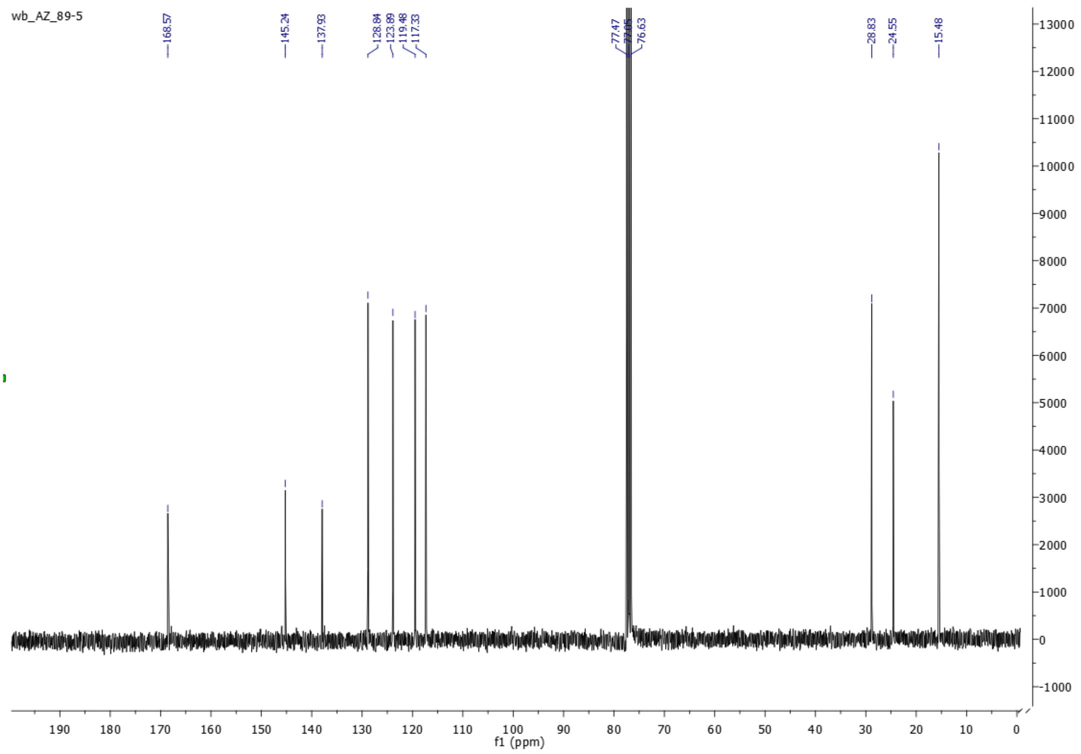

Figure S37. ^{13}C-NMR of compound **2i** isolated from the preparative scale bioacylation.

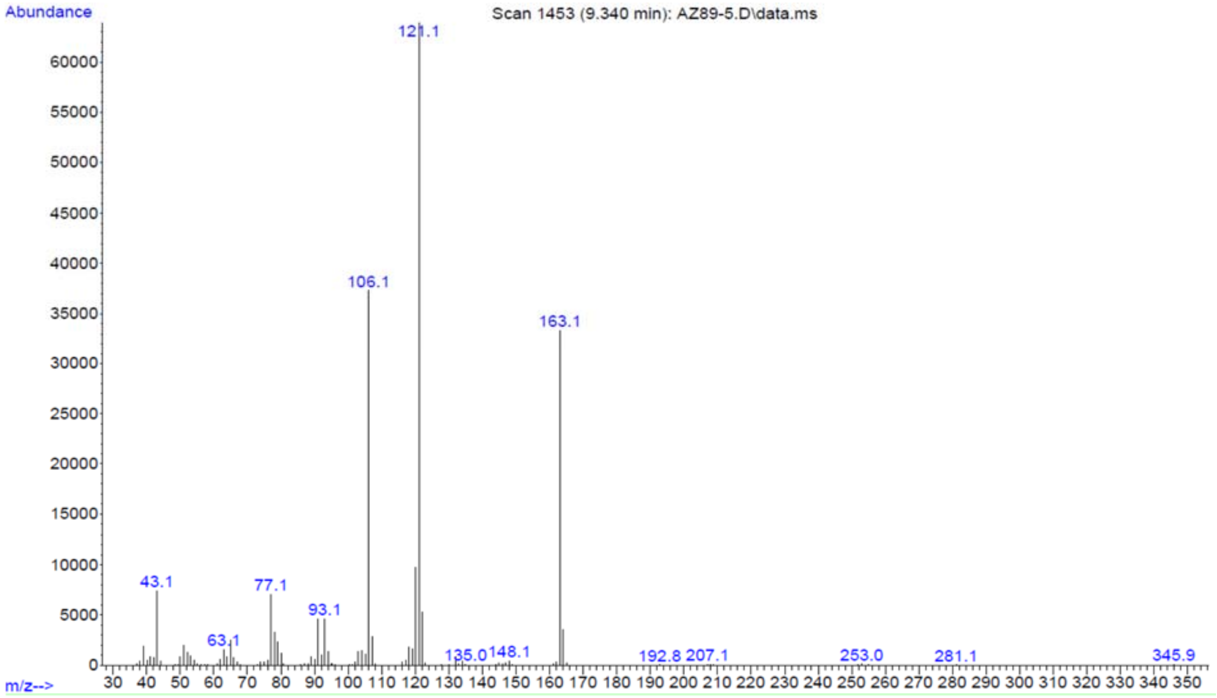

Figure S38. MS of compound **2i** isolated from the preparative scale bioacylation.

N-(2-ethylphenyl)acetamide (2j)

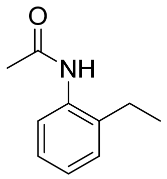

N-(2-ethylphenyl)acetamide was chemically obtained as a white solid with 50 % product yield. ^{1}H-NMR (300 MHz, $CDCl_3$): δ [ppm] = 1.23 (t, *J* = 7.6 Hz, 3 H), 2.19 (s, 3H), 2.48-2.71 (m, 2H). 7.10-7.35 (m, 4H), 7.69 (d, *J* = 7.6 Hz, 1H, N<u>H</u>), ^{13}C-NMR (75 MHz, $CDCl_3$): δ_C [ppm] = 14.0, 24.2, 24.2, 124.4, 125.8, 126.6, 128.5, 135.3, 135.7, 168.7.

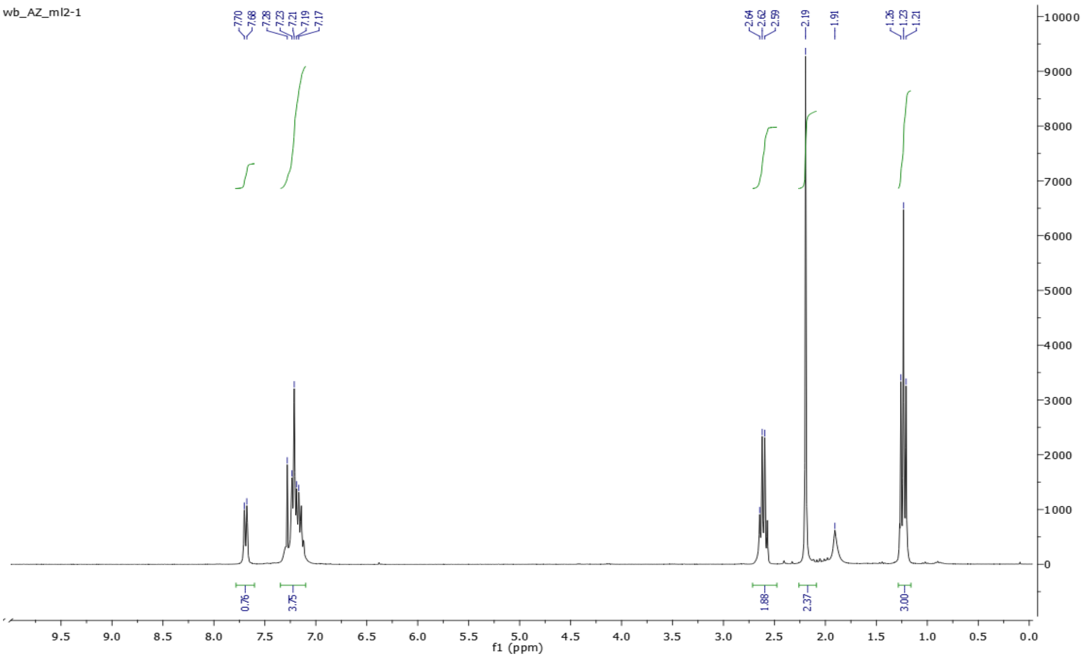

Figure S39. ^{1}H-NMR of compound **2j**.

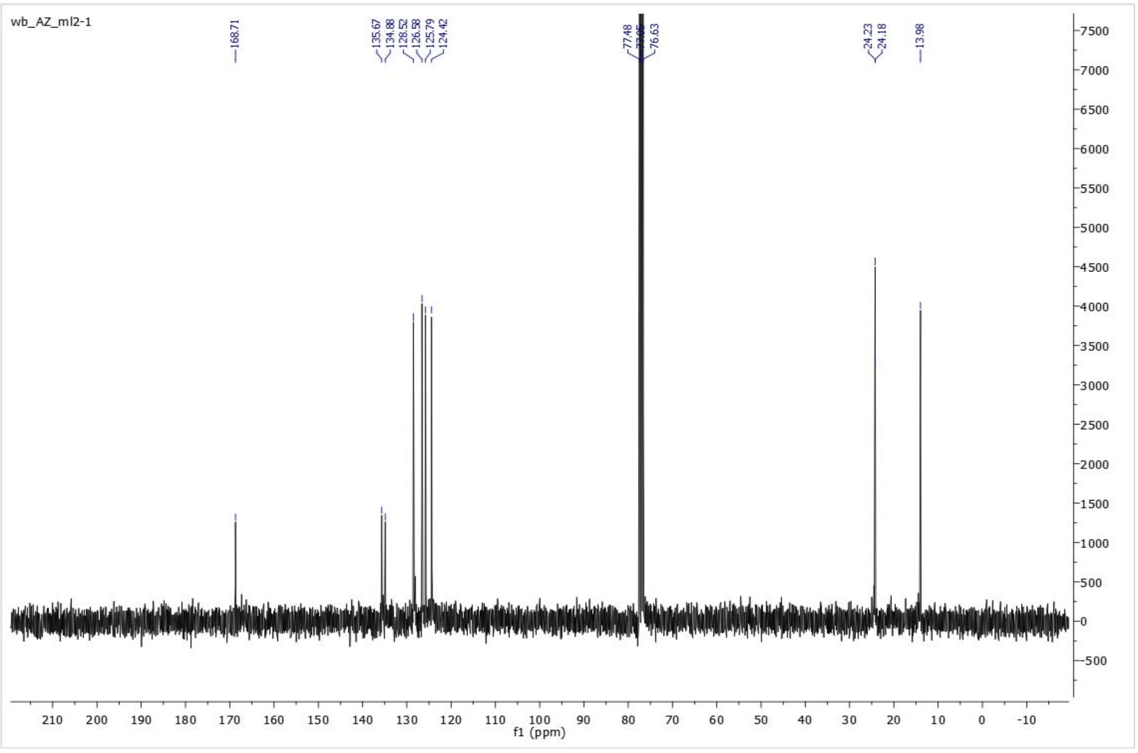

Figure S40. ^{13}C-NMR of compound **2j**.

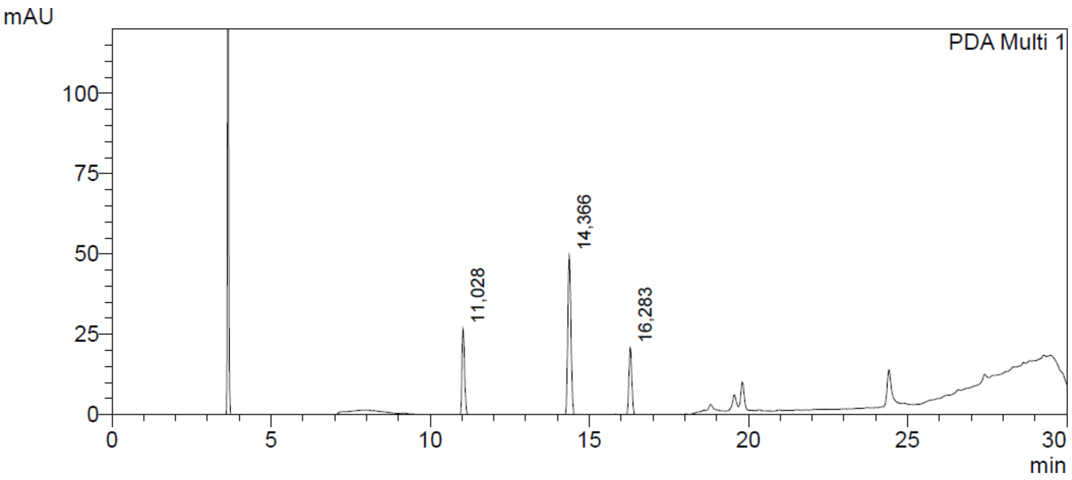

Figure S41. HPLC-chromatogram showing analytical-scale *N*-acylation of **1j** into **2j** (t_r = 16.3 min) using PA (t_r = 14.4 min) as an acyl donor.

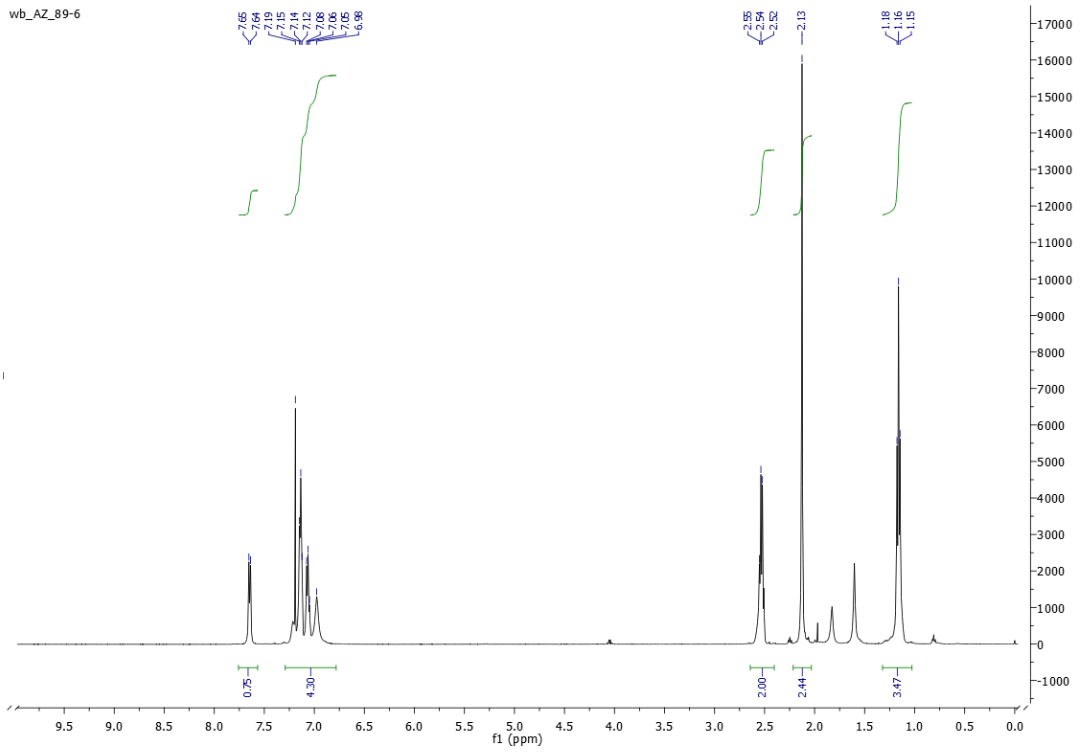

Figure S42. ^{1}H-NMR of compound **2j** isolated from the preparative scale bioacetylation.

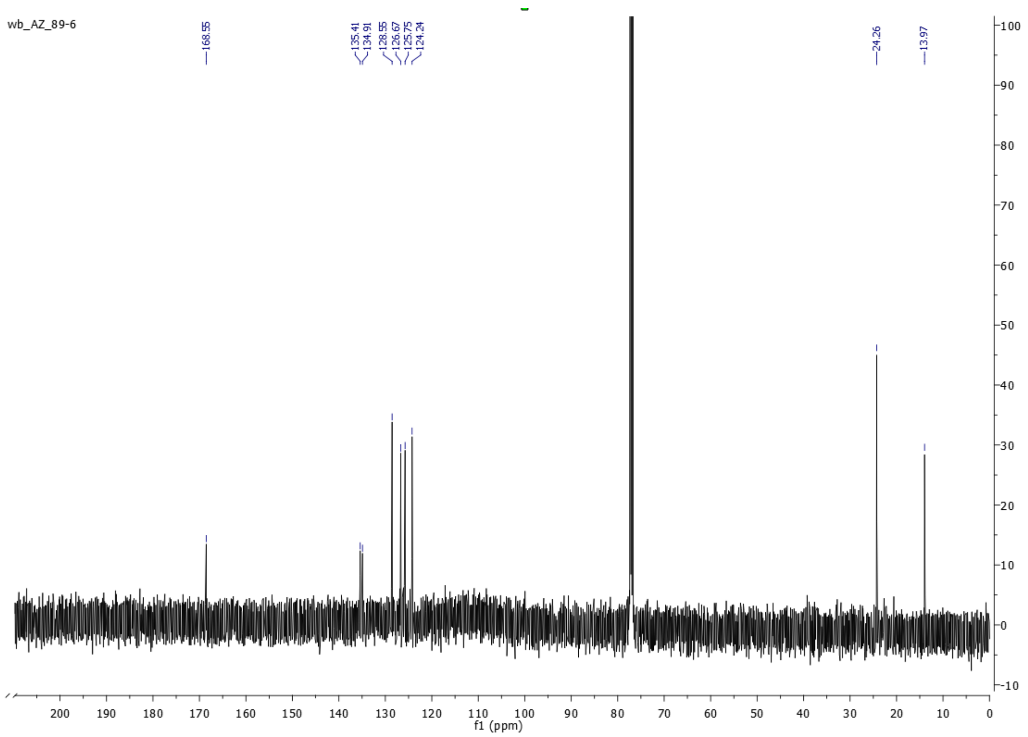

Figure S43. ^{13}C-NMR of compound **2j** isolated from the preparative scale bioacylation.

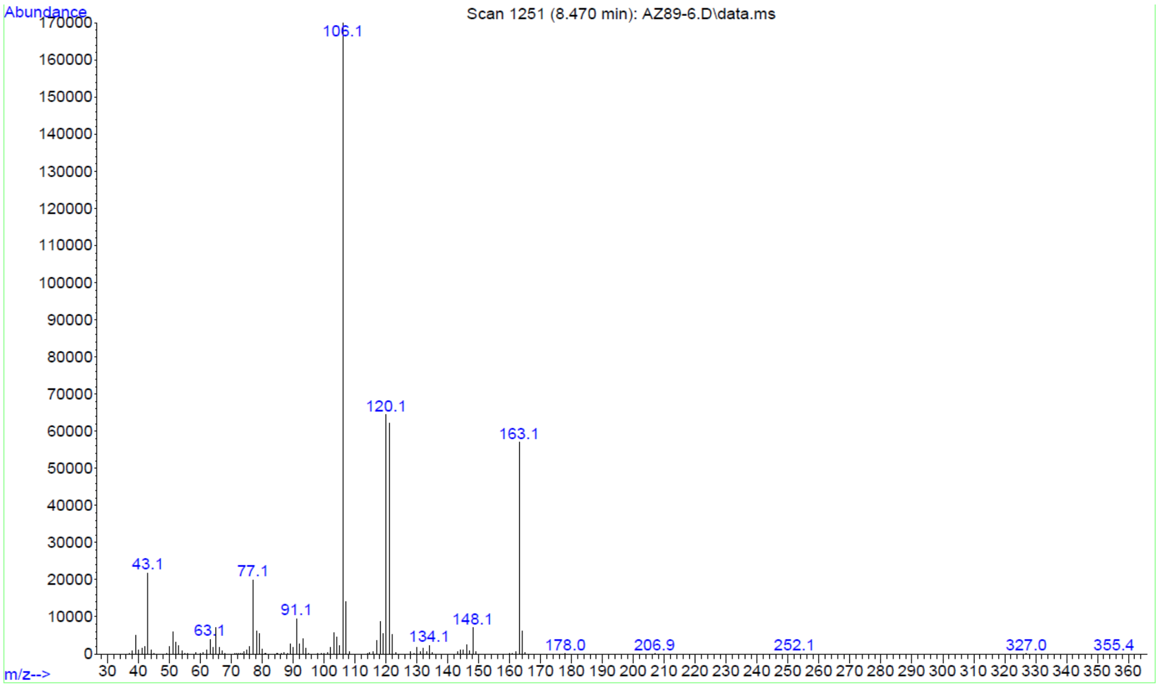

Figure S44. MS of compound **2j** isolated from the preparative scale bioacylation.

N-(4-chloro-3-(hydroxymethyl)phenyl)acetamide (2l)

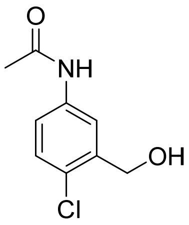

N-(4-chloro-3-(hydroxymethyl)phenyl)acetamide was chemically obtained as a pale yellow solid with 36 % product yield. ^{1}H-NMR (300 MHz, DMSO-d_6): δ [ppm] = 2.01 (s, 3 H), 4.51 (s, J = 5.4 Hz, 2H), 5.43 (t, J = 5.6 Hz, 1H). 7.29 (d, J = 8.6 Hz, 1H), 7.58 (dd, J_1 = 8.6 Hz, J_2 = 2.6 Hz, 1H), 7.72 (d, J = 2.5 Hz, 1H), 10.06 (s, 1H); ^{13}C-NMR (75 MHz, DMSO-d_6): δ_C [ppm] = 24.4, 60.3, 118.8, 118.9, 124.6, 129.3, 138.8, 140.3, 168.8.

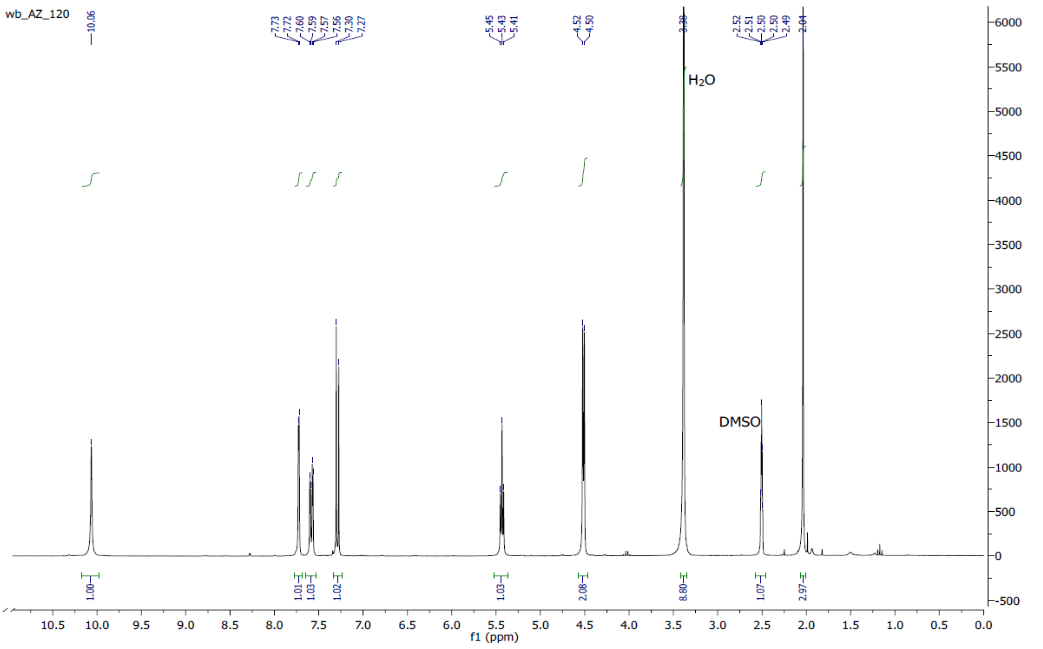

Figure S45. ^{1}H-NMR of compound **2l**.

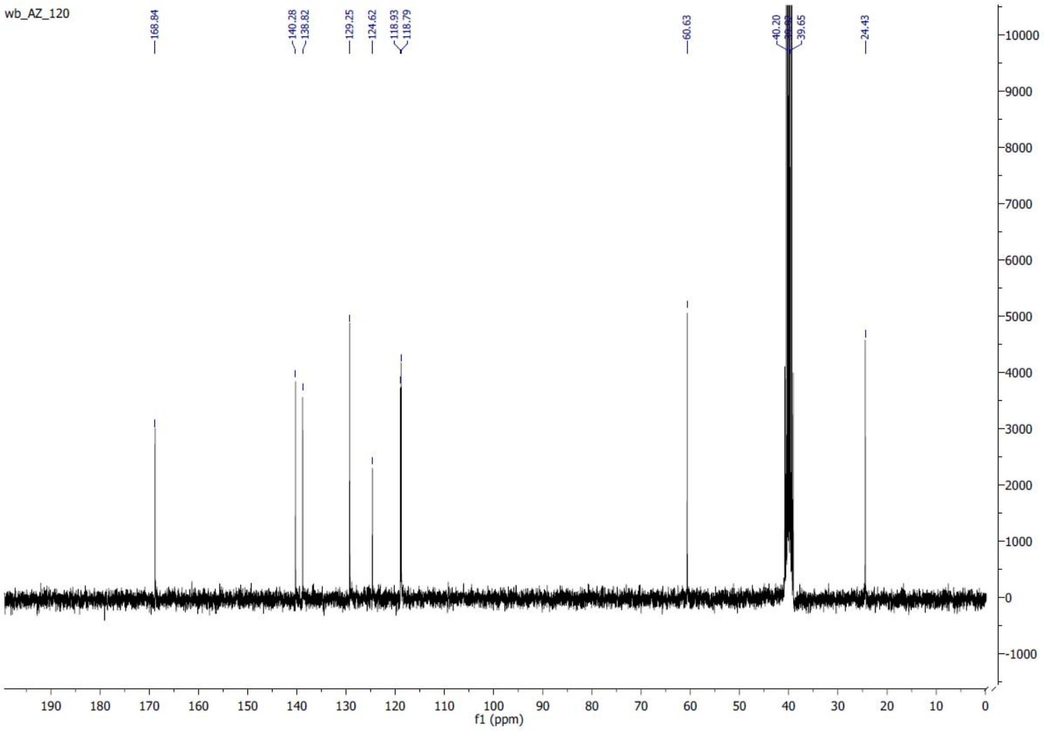

Figure S46. ^{13}C-NMR of compound **2l**.

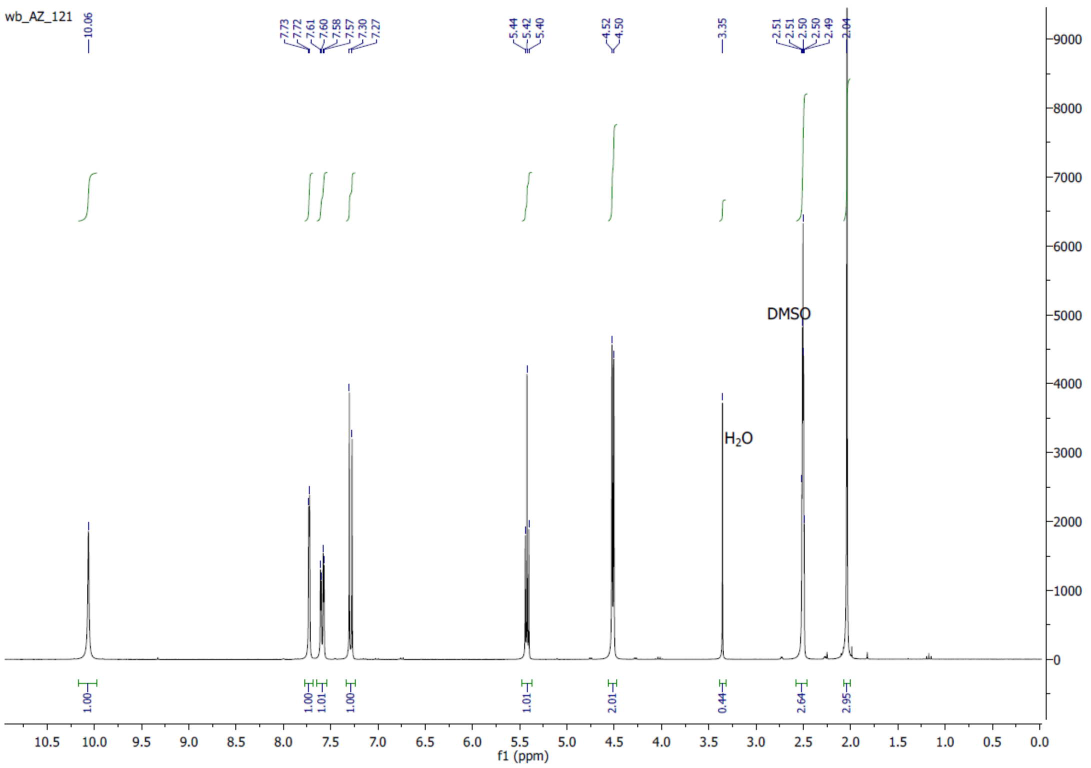

Figure S47. ^{1}H-NMR of compound **2l** isolated from the preparative scale bioacylation.

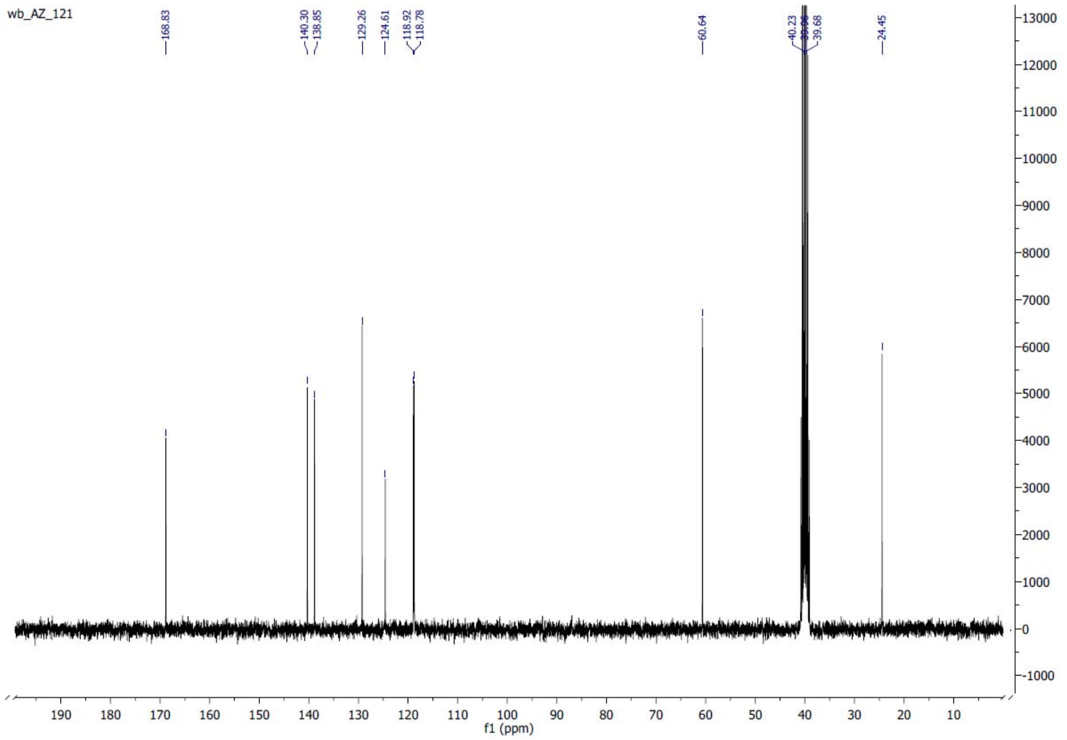

Figure S48. ^{13}C-NMR of compound **2l** isolated from the preparative scale bioacetylation.

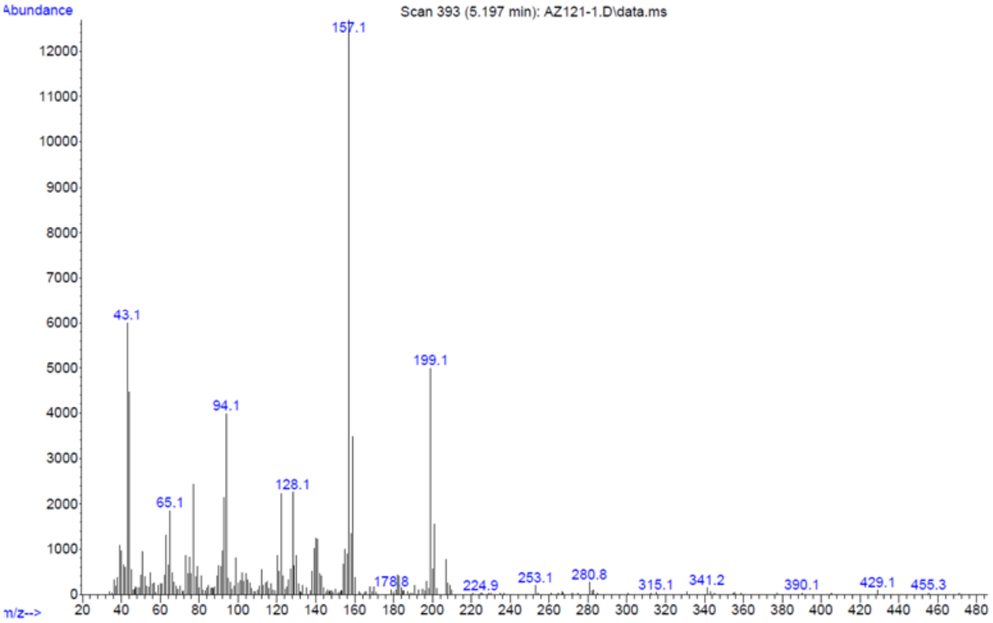

Figure S49. MS of compound **2l** isolated from the preparative scale bioacetylation.

a)

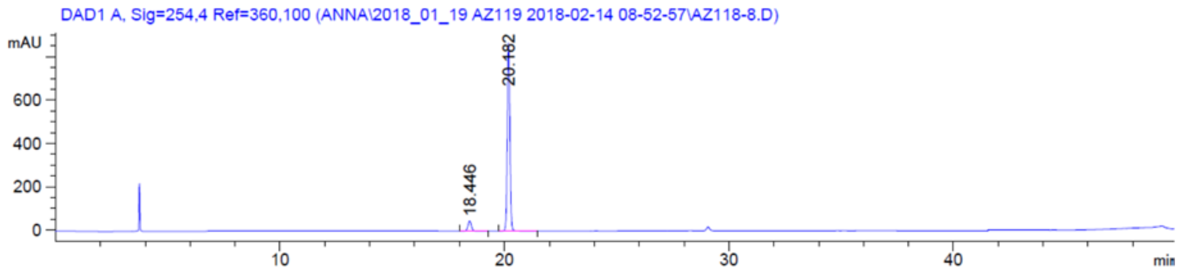

b)

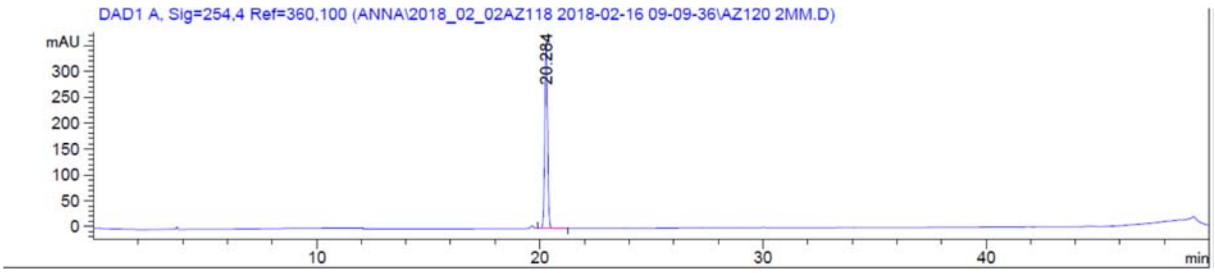

Figure S50. a) HPLC-chromatogram showing analytical-scale *N*-acetylation of **1l** into **2l** (t_r = 20.2 min) using PA (t_r = 18.4 min) as an acetyl donor; b) HPLC-chromatogram showing reference compound **2l** (t_r = 20.3 min). The following gradient elution with H2O and MeCN (+TFA, 0.1 vol. %) was performed: 0-15 % MeCN (0-10 min), 15-60 % MeCN (10-42 min), 60-100 % MeCN (42-45 min), 100-0 % MeCN (45-50 min), flow rate = 1 mL min^{-1}, λ = 254 nm, injection vol. = 2 µL. Reaction products were quantified at 254 nm from the peak areas on the basis of standard curves with reference compounds.

a)

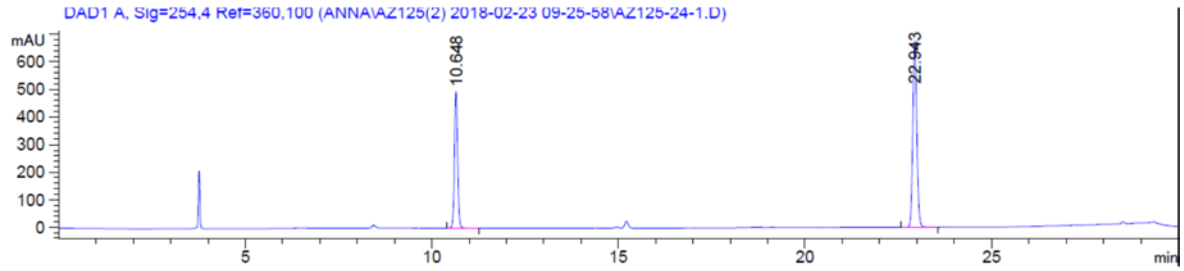

b)

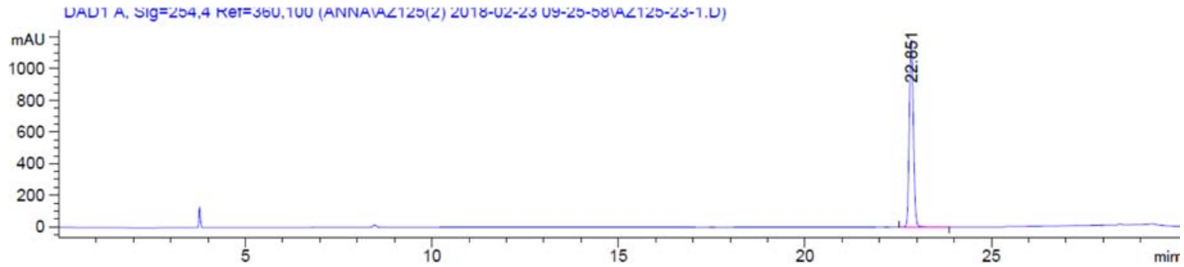

c)

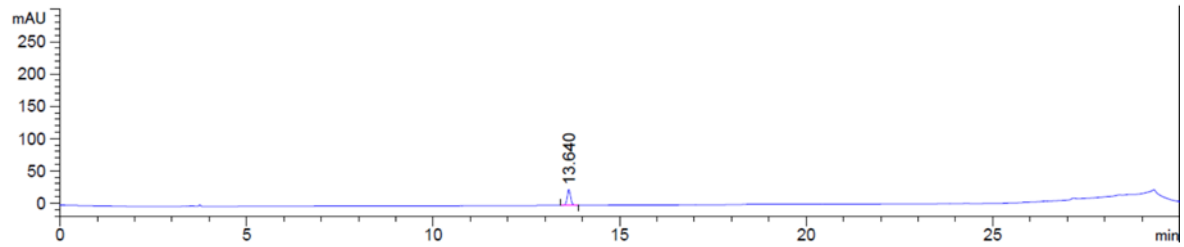

d)

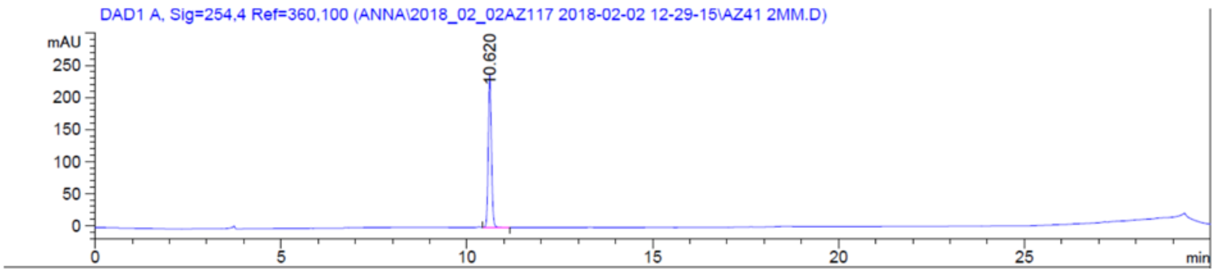

Figure S51. a) HPLC-chromatogram showing the bio-*N*-acetylation of **1a** and **1m** into **2a** (t_r = 10.6 min) and **2m** (t_r = 13.6 min) using DAPG (t_r = 22.9 min) as acetyl donor; b) HPLC-chromatogram showing acetylation of **1a** and **1m** using DAPG as acetyl donor without enzyme; c) HPLC-chromatogram of reference compound **2m** (t_r = 13.6 min); d) HPLC-chromatogram of reference compound **2a** (t_r = 10.6 min).

a)

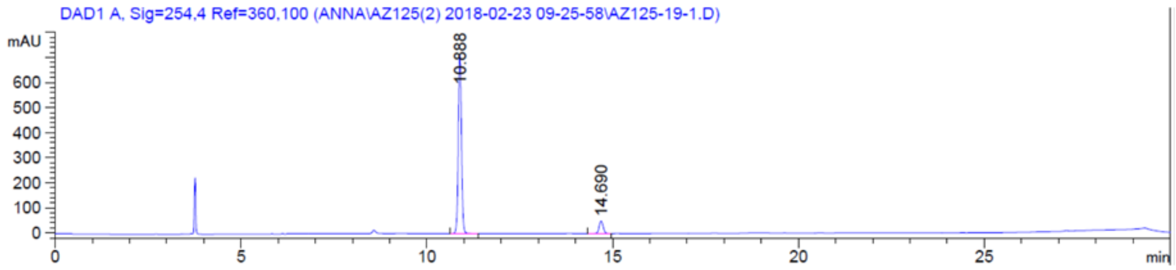

b)

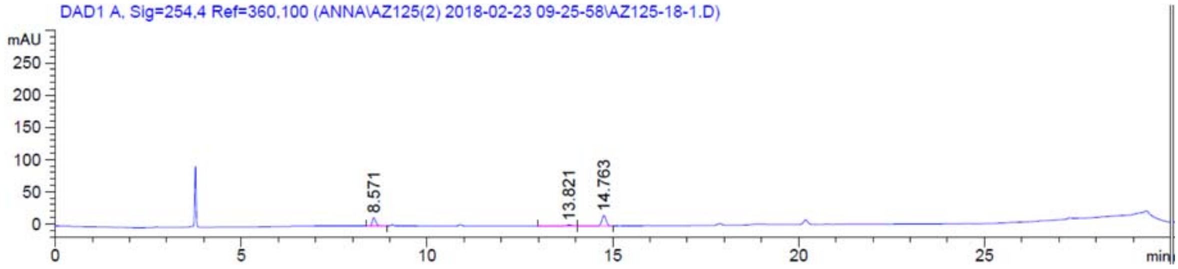

Figure S52. a) HPLC-chromatogram showing analytical-scale bio-*N*-acetylation of **1a** and **1m** into **2a** (t_r = 10.6 min) and **2m** (t_r = 13.8 min) using PA (t_r = 14.7 min) as acetyl donor; b) HPLC-chromatogram showing analytical-scale reaction of **1a** and **1m** using PA as acetyl donor in the absence of enzyme.

a)

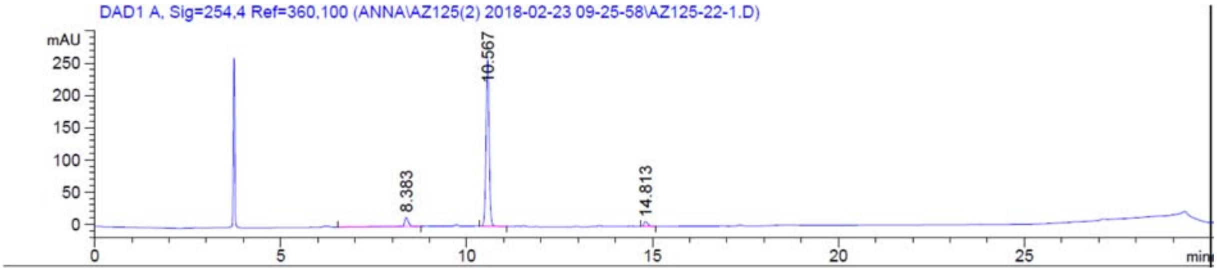

b)

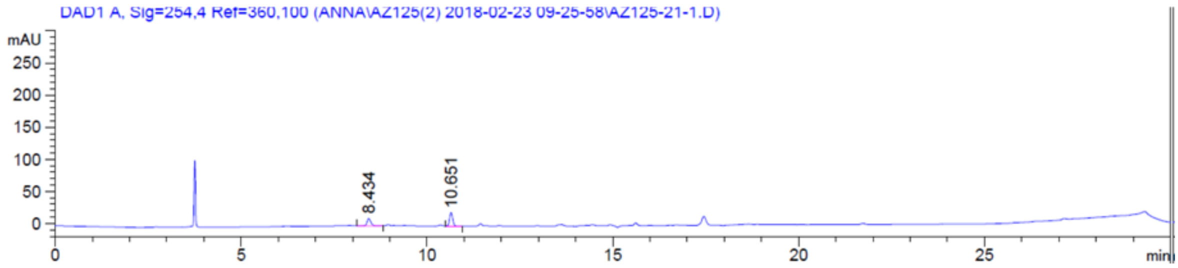

Figure S53. a) HPLC-chromatogram showing analytical-scale bio-*N*-acetylation of **1a** and **1m** into **2a** (t_r = 10.6 min) and **2m** (t_r = 13.6 min) using IPEA as acetyl donor; b) HPLC-chromatogram showing analytical-scale reaction of **1a** and **1m** using IPEA as acetyl donor in the absence of enzyme.

References

[1] (a) N. G. Schmidt, T. Pavkov-Keller, N. Richter, B. Wiltschi, K. Gruber, W. Kroutil, *Angew. Chem. Int. Ed.*, 2017, **56**, 7615-7619; (b) N. G. Schmidt, W. Kroutil, *Eur. J. Org. Chem.*, 2017, **39**, 5865-5871.

[2] A. Alalla, M. Merabet-Khelassi, L. Aribi-Zouioueche, O. Riant, *Synth. Comm.* **2014**, *44*, 2364-2376.

[3] A. Hayashi, H. Saitou, T. Mori, I. Matano, H. Sugisaki, K. Maruyama, *Biosci. Biotechnol. Biochem.* **2012**, *76*, 559-566.
